# A single-nucleus transcriptomic atlas of primate liver aging uncovers the pro-senescence role of SREBP2 in hepatocytes

**DOI:** 10.1093/procel/pwad039

**Published:** 2023-06-28

**Authors:** Shanshan Yang, Chengyu Liu, Mengmeng Jiang, Xiaoqian Liu, Lingling Geng, Yiyuan Zhang, Shuhui Sun, Kang Wang, Jian Yin, Shuai Ma, Si Wang, Juan Carlos Izpisua Belmonte, Weiqi Zhang, Jing Qu, Guang-Hui Liu

**Affiliations:** Advanced Innovation Center for Human Brain Protection and National Clinical Research Center for Geriatric Disorders, Xuanwu Hospital Capital Medical University, Beijing 100053, China; Aging Translational Medicine Center, International Center for Aging and Cancer, Beijing Municipal Geriatric Medical Research Center, Xuanwu Hospital, Capital Medical University, Beijing 100053, China; Xuanwu Hospital Capital Medical University, Beijing 100053, China; State Key Laboratory of Stem Cell and Reproductive Biology, Institute of Zoology, Chinese Academy of Sciences, Beijing 100101, China; University of Chinese Academy of Sciences, Beijing 100049, China; State Key Laboratory of Membrane Biology, Institute of Zoology, Chinese Academy of Sciences, Beijing 100101, China; Institute for Stem Cell and Regeneration, Chinese Academy of Sciences, Beijing 100101, China; Beijing Institute for Stem Cell and Regenerative Medicine, Beijing 100101, China; State Key Laboratory of Stem Cell and Reproductive Biology, Institute of Zoology, Chinese Academy of Sciences, Beijing 100101, China; Institute for Stem Cell and Regeneration, Chinese Academy of Sciences, Beijing 100101, China; Beijing Institute for Stem Cell and Regenerative Medicine, Beijing 100101, China; Advanced Innovation Center for Human Brain Protection and National Clinical Research Center for Geriatric Disorders, Xuanwu Hospital Capital Medical University, Beijing 100053, China; Aging Translational Medicine Center, International Center for Aging and Cancer, Beijing Municipal Geriatric Medical Research Center, Xuanwu Hospital, Capital Medical University, Beijing 100053, China; State Key Laboratory of Membrane Biology, Institute of Zoology, Chinese Academy of Sciences, Beijing 100101, China; Beijing Institute for Stem Cell and Regenerative Medicine, Beijing 100101, China; State Key Laboratory of Membrane Biology, Institute of Zoology, Chinese Academy of Sciences, Beijing 100101, China; Institute for Stem Cell and Regeneration, Chinese Academy of Sciences, Beijing 100101, China; Beijing Institute for Stem Cell and Regenerative Medicine, Beijing 100101, China; State Key Laboratory of Membrane Biology, Institute of Zoology, Chinese Academy of Sciences, Beijing 100101, China; University of Chinese Academy of Sciences, Beijing 100049, China; State Key Laboratory of Membrane Biology, Institute of Zoology, Chinese Academy of Sciences, Beijing 100101, China; University of Chinese Academy of Sciences, Beijing 100049, China; State Key Laboratory of Membrane Biology, Institute of Zoology, Chinese Academy of Sciences, Beijing 100101, China; Institute for Stem Cell and Regeneration, Chinese Academy of Sciences, Beijing 100101, China; Beijing Institute for Stem Cell and Regenerative Medicine, Beijing 100101, China; Advanced Innovation Center for Human Brain Protection and National Clinical Research Center for Geriatric Disorders, Xuanwu Hospital Capital Medical University, Beijing 100053, China; Aging Translational Medicine Center, International Center for Aging and Cancer, Beijing Municipal Geriatric Medical Research Center, Xuanwu Hospital, Capital Medical University, Beijing 100053, China; Altos Labs, Inc., San Diego, CA 94022, USA; CAS Key Laboratory of Genomic and Precision Medicine, Beijing Institute of Genomics, Chinese Academy of Sciences and China National Center for Bioinformation, Beijing 100101, China; University of Chinese Academy of Sciences, Beijing 100049, China; Institute for Stem Cell and Regeneration, Chinese Academy of Sciences, Beijing 100101, China; Aging Biomarker Consortium, Beijing 100101, China; State Key Laboratory of Stem Cell and Reproductive Biology, Institute of Zoology, Chinese Academy of Sciences, Beijing 100101, China; University of Chinese Academy of Sciences, Beijing 100049, China; Institute for Stem Cell and Regeneration, Chinese Academy of Sciences, Beijing 100101, China; Beijing Institute for Stem Cell and Regenerative Medicine, Beijing 100101, China; Aging Biomarker Consortium, Beijing 100101, China; Advanced Innovation Center for Human Brain Protection and National Clinical Research Center for Geriatric Disorders, Xuanwu Hospital Capital Medical University, Beijing 100053, China; State Key Laboratory of Membrane Biology, Institute of Zoology, Chinese Academy of Sciences, Beijing 100101, China; Aging Translational Medicine Center, International Center for Aging and Cancer, Beijing Municipal Geriatric Medical Research Center, Xuanwu Hospital, Capital Medical University, Beijing 100053, China; Xuanwu Hospital Capital Medical University, Beijing 100053, China; University of Chinese Academy of Sciences, Beijing 100049, China; Institute for Stem Cell and Regeneration, Chinese Academy of Sciences, Beijing 100101, China; Beijing Institute for Stem Cell and Regenerative Medicine, Beijing 100101, China; Aging Biomarker Consortium, Beijing 100101, China

**Keywords:** single-nucleus RNA sequencing, liver, hepatocytes, aging, senescence, SREBP2

## Abstract

Aging increases the risk of liver diseases and systemic susceptibility to aging-related diseases. However, cell type-specific changes and the underlying mechanism of liver aging in higher vertebrates remain incompletely characterized. Here, we constructed the first single-nucleus transcriptomic landscape of primate liver aging, in which we resolved cell type-specific gene expression fluctuation in hepatocytes across three liver zonations and detected aberrant cell–cell interactions between hepatocytes and niche cells. Upon in-depth dissection of this rich dataset, we identified impaired lipid metabolism and upregulation of chronic inflammation-related genes prominently associated with declined liver functions during aging. In particular, hyperactivated sterol regulatory element-binding protein (SREBP) signaling was a hallmark of the aged liver, and consequently, forced activation of SREBP2 in human primary hepatocytes recapitulated *in vivo* aging phenotypes, manifesting as impaired detoxification and accelerated cellular senescence. This study expands our knowledge of primate liver aging and informs the development of diagnostics and therapeutic interventions for liver aging and associated diseases.

## Introduction

Medical care advances, improved hygiene, and better access to food and water have resulted in world-wide population aging, which in and of itself has unavoidable global ramifications. Because a longer life span does not necessarily mean a longer healthy life, the number of elderly individuals with chronic diseases, including metabolic and cardiovascular diseases, has increased dramatically (Costantino et al., 2016; Maeso-Díaz and Gracia-Sancho, 2020; Cai et al., 2022). As the largest solid and metabolic organ, the liver is a nexus for homeostatic maintenance throughout the human body (Rhyu and Yu, 2021). The liver performs a wide range of physiological functions, such as energy metabolism and storage, molecular biosynthesis, and scavenging of xenobiotics (Hunt et al., 2019; He et al., 2022). Consequently, liver aging not only increases its vulnerability to acute injury and liver diseases but also promotes susceptibility to the inflammatory and fibrotic responses that are associated with systemic aging-related diseases, such as diabetes and aging-related cardiometabolic diseases (Ahmadieh and Azar, 2014; Kohsari et al., 2021; van der Meer et al., 2022). Given these relationships, the field of liver aging research is actively growing.

Aging-associated changes in the liver include volume loss, decline of blood flow, increase in inflammatory response, accumulation of senescent cells, and progressive organ dysfunction (Hunt et al., 2019; Shen et al., 2022). Many extrinsic and intrinsic factors are known to contribute to liver aging, including genomic and epigenomic alterations as well as dysregulation in mitochondrial function or nutrient-sensing pathways (Sastre et al., 2007; Mann, 2014; Hunt et al., 2019). Based on studies in rodents, several aging-related pathophysiological phenotypes in the liver have been identified. These include the accumulation of polyploid nuclei and DNA damage marks in hepatocytes of aged mice (Maslov et al., 2013; Silva et al., 2018) and the increased thickness of the liver sinusoidal endothelial cells (LSEC) layer, with the number and size of fenestrations reduced (Le Couteur et al., 2007; Poisson et al., 2017). At a cellular level, hepatic stellate cells (HSC) are hyperactivated, leading to the development of hepatic fibrosis with age (Krizhanovsky et al., 2008; Warren et al., 2011; Puche et al., 2013), and the number of resident macrophages in the liver, the Kupffer cells, also increases and becomes aberrantly activated during aging (Dixon et al., 2013; Stahl et al., 2018). Thus, there is irrefutable evidence that aging has a profound impact on different cell types in the liver. However, the molecular mechanisms associated with the cell type-specific aging phenotypes in the liver and their mutual crosstalk remain poorly understood. Therefore, an in-depth exploration of liver aging stands to enhance our knowledge about how aging impacts liver cellular composition and gene expression.

Single-cell RNA sequencing (scRNA-seq) and single-nucleus RNA sequencing (snRNA-seq) have been leveraged to elucidate the transcriptomic landscape for liver aging and its associated diseases, such as hepatocellular carcinoma (HCC), cirrhosis, liver fibrosis, and non-alcoholic steatohepatitis (Krenkel et al., 2019; Ramachandran et al., 2019; Ho et al., 2021; Wang et al., 2021b; Barreby et al., 2022; Hundertmark et al., 2022; Zhou et al., 2022; Zou et al., 2022). Rodents are commonly used as the canonical model for this kind of research. However, there are many profound differences between rodents and primates, including the presence of lobated architecture in the livers of rodents but not in primates, and a lower amount of connective tissue in the portal tracts of rodents compared to primates (Vons et al., 2009; Kruepunga et al., 2019). Rodents also have a higher percentage of polyploid hepatocytes than primates (Donne et al., 2021). Furthermore, large mammals are more dependent on the hepatic artery for lobular perfusion than rodents (Kruepunga et al., 2019). Finally, expression levels and catalytic activities, and isoform composition of drug-metabolizing enzymes are also species-specific (Danek et al., 1988; Martignoni et al., 2006). For example, cytochrome P450 proteins (CYPs), the main enzymes involved in oxidative reactions and playing important roles as scavengers of xenobiotics, differ significantly between species (Villeneuve and Pichette, 2004; Martignoni et al., 2006). Thus, to investigate the biology of human liver aging, it is most relevant to do so in non-human primates (NHPs), whose liver structures, cellular composition, and biochemical functions are more similar to those of humans than those of rodents.

Here, we performed snRNA-seq, which permitted an unbiased characterization of major cell types in frozen liver samples, to conduct a thorough analysis of transcriptomic changes during liver aging at single-cell resolution. The hepatocyte itself was the main target cell type impacted by aging, as reflected by disordered glucose and lipid metabolism in aged livers. During aging, the interactions between hepatocytes and niche cell types, such as hepatic stellate cells and Kupffer cells, became elevated, which was reflected in a mutual activation of series of signaling pathways, including transforming growth factor-β (TGFβ) and interleukin (IL) signaling, likely to result in increased fibrosis, inflammation, and functional disorders in aged livers. As the fundamental driving force of changes in hepatocytes during liver aging, we identified sterol regulatory element-binding protein 2 (*SREBP2*, alias *SREBF2*), which functions as an upstream broad regulon for aging-associated differentially expressed genes (DEGs) in all zonation subtypes of hepatocytes, as inferred by gene regulatory networks based on co-expression and motif enrichment analysis. Furthermore, the activated form of SREBP2 protein was increased in aged livers. Consistently, in human primary hepatocytes, we demonstrated that the activated SREBP2 was capable of triggering senescence, mirroring our *in vivo* observations, and indicating its potential as an intervention target for liver aging and aging-related diseases.

## Results

### Histological features of liver aging in cynomolgus monkeys

**Figure 1.**
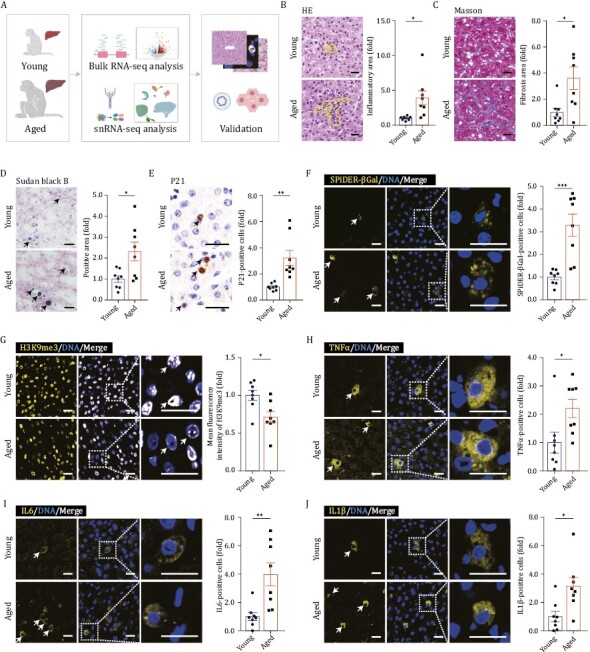
**Histological features of liver aging in cynomolgus monkeys.** (A) Schematic diagram illustrating sample collection, data analyses and validation. “Young” denotes cynomolgus monkeys 4–6 years old; “Aged” denotes cynomolgus monkeys 18–21 years old. Created with BioRender.com. (B) HE staining in liver tissues from young and aged monkeys. Representative images are shown on the left; quantitative data for the area of inflammatory focus are shown on the right. Scale bars, 25 μm. (C) Masson’s trichrome staining in liver tissues from young and aged monkeys. Representative images are shown on the left; quantitative data for the positive area of fibrosis are shown on the right. Scale bars, 25 μm. (D) Sudan Black B staining in liver tissues from young and aged monkeys. Representative images are shown on the left; quantitative data for the positive area of the Sudan Black B are shown on the right. Scale bars, 25 μm. (E) Immunohistochemistry staining of P21 in liver tissues from young and aged monkeys. Representative images are shown on the left; quantitative data for the percentage of P21-positive cells are shown on the right. Scale bars, 25 μm. (F) Immunofluorescence staining of SPiDER-βGal in liver tissues from young and aged monkeys. Representative images are shown on the left; quantitative data for the percentage of SPiDER-βGal-positive cells are shown on the right. Scale bars, 20 μm. (G) Immunofluorescence staining of H3K9me3 in liver tissues from young and aged monkeys. Representative images are shown on the left; quantitative data for the mean fluorescence intensity of H3K9me3 are shown on the right. Scale bars, 20 μm. (H) Immunofluorescence staining of TNFα in liver tissues from young and aged monkeys. Representative images are shown on the left; quantitative data for the percentage of TNFα-positive cells are shown on the right. Scale bars, 20 μm. (I) Immunofluorescence staining of IL6 in liver tissues from young and aged monkeys. Representative images are shown on the left; quantitative data for the percentage of IL6-positive cells are shown on the right. Scale bars, 20 μm. (I) Immunofluorescence staining of IL1β in liver tissues from young and aged monkeys. Representative images are shown on the left; quantitative data for the percentage of IL1β-positive cells are shown on the right. Scale bars, 20 μm. (B–J) Data are quantified as fold changes and shown as means ± SEM. Young, *n* = 8 monkeys; aged, *n* = 8 monkeys. ^*^P < 0.05, ^**^P < 0.01, ^***^P < 0.001.

To determine aging-related changes in primates, we obtained liver tissues from eight young (4–6 years old) and eight aged (18–21 years old) cynomolgus monkeys, respectively, equivalent to teenagers and elderly adults (people in their seventies) (Figs. 1A and S1A) (Wang et al., 2020b; Zhang et al., 2020, 2021; Huang et al., 2022). Between the cohorts, body mass index (BMI) trended upward while the liver-to-body weight ratio decreased in elderly adult primates (Fig. S1B and S1C). By histochemical analyses, we observed chronic inflammation manifested by increased infiltrating immune cells in aged livers (Fig. 1B). In addition, fibrosis in the parenchymal regions of the aged liver was increased relative to that of its younger counterparts (Fig. 1C). Moreover, by using Sudan black B staining, we found that lipofuscin, a kind of oxidized lipid drops, accumulated both within and between cells in the liver tissues of aged monkeys (Fig. 1D). Lipofuscin is known to accumulate in senescent cells as a senescence indicator (Evangelou and Gorgoulis, 2017). Consistently, we observed higher proportions of P21-positive cells and SPiDER-βGal-positive cells and attenuated signals for heterochromatin mark H3K9me3 in aged livers (Fig. 1E–G), together indicating aggravated cellular senescence in aged livers (Ma et al., 2021; Zhang et al., 2021; Liu et al., 2023). In line with the increased accumulation of senescent cells, we also noticed that senescence-associated secretory phenotype (SASP)-associated factors (Sun et al., 2022), including tumor necrosis factor alpha (TNFα), interleukin-6 (IL6) and interleukin-1β (IL1β), were specifically upregulated in aged livers (Fig. 1H–J). Taken together, although the overall appearance of aged livers was barely indistinguishable from that of young livers, their phenotype based on the histological analysis was degenerated.

### Transcriptomic features of liver aging in cynomolgus monkeys

**Figure 2.**
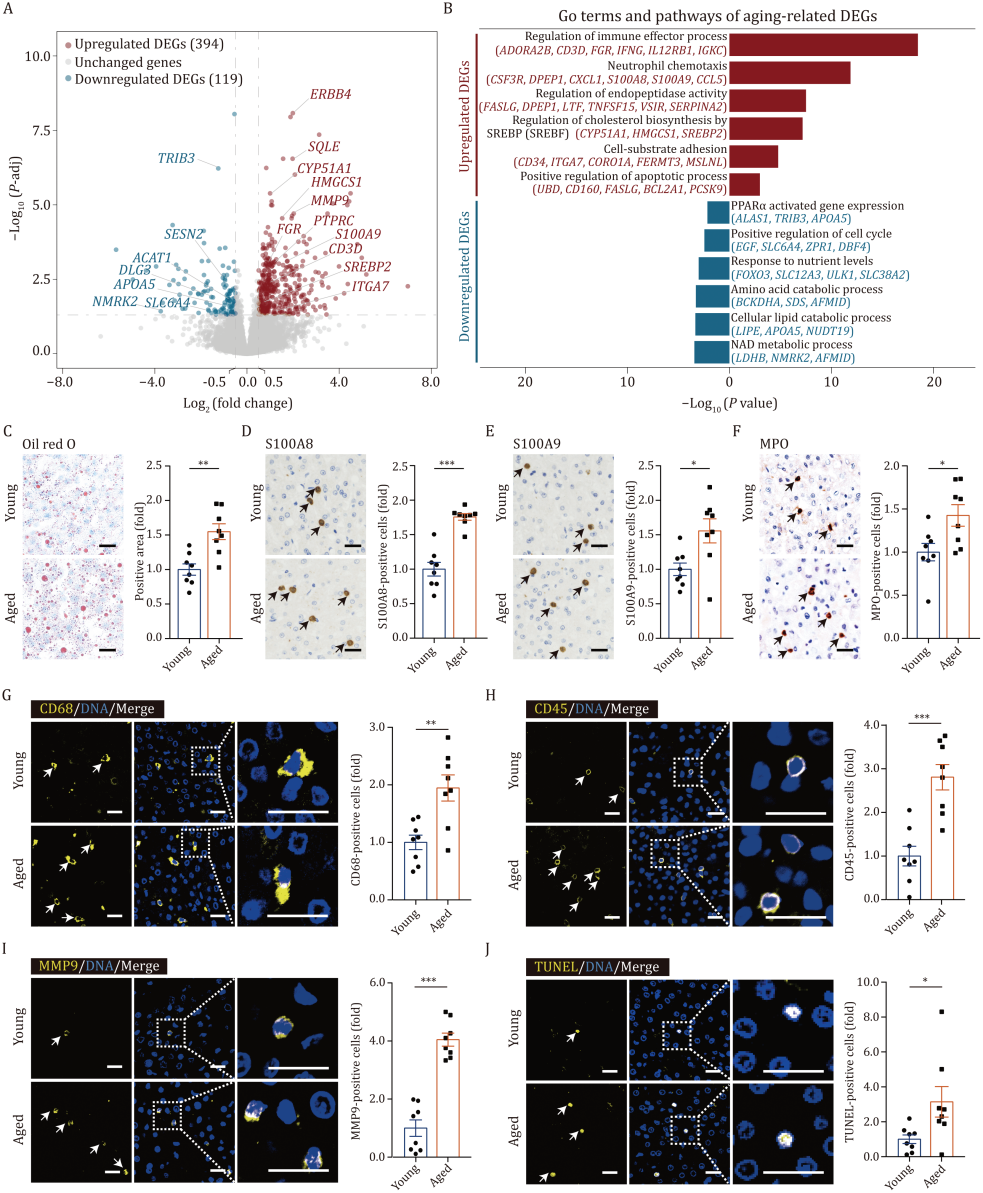
**Transcriptomic features of liver aging in cynomolgus monkeys.** (A) Volcano plot shows differentially expressed genes (DEGs) of bulk RNA-seq between aged and young livers (aged/young). Red points represent upregulated DEGs; blue points represent downregulated DEGs; gray points represent unchanged genes. (B) Representative GO terms and pathways of upregulated and downregulated DEGs in aged and young monkey livers. (C) Oil red O staining in liver tissues from young and aged monkeys. Representative images are shown on the left; quantitative data for the positive area of the oil red O staining are shown on the right. Scale bars, 25 μm. (D) Immunohistochemistry staining of S100A8 in liver tissues from young and aged monkeys. Representative images are shown on the left; quantitative data for the percentage of S100A8-positive cells are shown on the right. Scale bars, 25 μm. (E) Immunohistochemistry staining of S100A9 in liver tissues from young and aged monkeys. Representative images are shown on the left; quantitative data for the percentage of S100A9-positive cells are shown on the right. Scale bars, 25 μm. (F) Immunohistochemistry staining of MPO in liver tissues from young and aged monkeys. Representative images are shown on the left; quantitative data for the percentage of MPO-positive cells are shown on the right. Scale bars, 25 μm. (G) Immunofluorescence staining of CD68 in liver tissues from young and aged monkeys. Representative images are shown on the left; quantitative data for the percentage of CD68-positive cells are shown on the right. Scale bars, 20 μm. (H) Immunofluorescence staining of CD45 in liver tissues from young and aged monkeys. Representative images are shown on the left; quantitative data for the percentage of CD45-positive cells are shown on the right. Scale bars, 20 μm. (I) Immunofluorescence staining of MMP9 in liver tissues from young and aged monkeys. Representative images are shown on the left; quantitative data for the percentage of MMP9-positive cells are shown on the right. Scale bars, 20 μm. (J) TUNEL staining in liver tissues from young and aged monkeys. Representative images are shown on the left; quantitative data for the percentage of TUNEL-positive cells are shown on the right. Scale bars, 20 μm. (C–J) Data are quantified as fold changes and shown as means ± SEM. Young, *n* = 8 monkeys; aged, *n* = 8 monkeys. ^*^P < 0.05, ^**^P < 0.01, ^***^P < 0.001.

To generate a global view of the transcriptional response to aging in the liver, we conducted bulk RNA sequencing (bulk RNA-seq) in young and aged livers. In total, we identified 394 upregulated and 119 downregulated aging-associated DEGs (adjusted *P* value ≤ 0.05, | Log_2_ (fold change), Log_2_FC | ≥ 0.5) by bulk RNA-seq (Figs. 2A, S1D, S1E and Table S1). In aged livers, genes related to lipid and energy metabolism were downregulated, such as peroxisome proliferator-activated receptor α (PPARα) activated gene expression, and cellular lipid catabolic process (Fig. 2B). Dysfunction of the lipid and fatty acid catabolic process could lead to accumulation of lipid droplets (Zhou et al., 2018; Shen et al., 2022), which was confirmed by Oil red O staining in monkey livers (Fig. 2C). Response to nutritional stress is critical for the maintaining metabolic homeostasis and viability (Russell et al., 2014), its downregulation in the aged liver may induce homeostatic imbalance in hepatic metabolic function (Fig. 2B). In contrast, neutrophil chemotaxis, regulation of immune effector process, endopeptidase activity, and cholesterol biosynthesis by SREBP2, cell-substrate adhesion and positive regulation of apoptotic process were upregulated (Fig. 2B). In line with the transcriptional changes, genes encoding alarmin S100A8/S100A9, a kind of damage-associated molecular patterns (DAMP) factors, were upregulated in aged livers as indicated in immunohistochemistry results (Fig. 2D and 2E). Concomitantly, we detected increased proportions of neutrophils by immunostaining of their marker myeloperoxidase (MPO) (Fig. 2F). Besides, the accumulation of macrophages was observed by immunostaining of their marker CD68 (Fig. 2G), and a nearly 3-fold increase in immune cells was identified by CD45 immunostaining (Fig. 2H), both indications of an increased immune response in aged livers. In addition, levels of MMP9, an endopeptidase involved in remodeling of the extracellular matrix (ECM), were also increased and verified experimentally (Fig. 2I). And we detected aggregation of apoptotic cells, as indicated by TUNEL-positive cells, in aged livers, consistent with the upregulated apoptotic process in bulk RNA-seq (Fig. 2J). Taken together, our comprehensive histological and transcriptomic analyses revealed remarkable aging-associated changes in primate livers.

### Single-nucleus transcriptomics identifies major cell types in cynomolgus monkey livers

**Figure 3.**
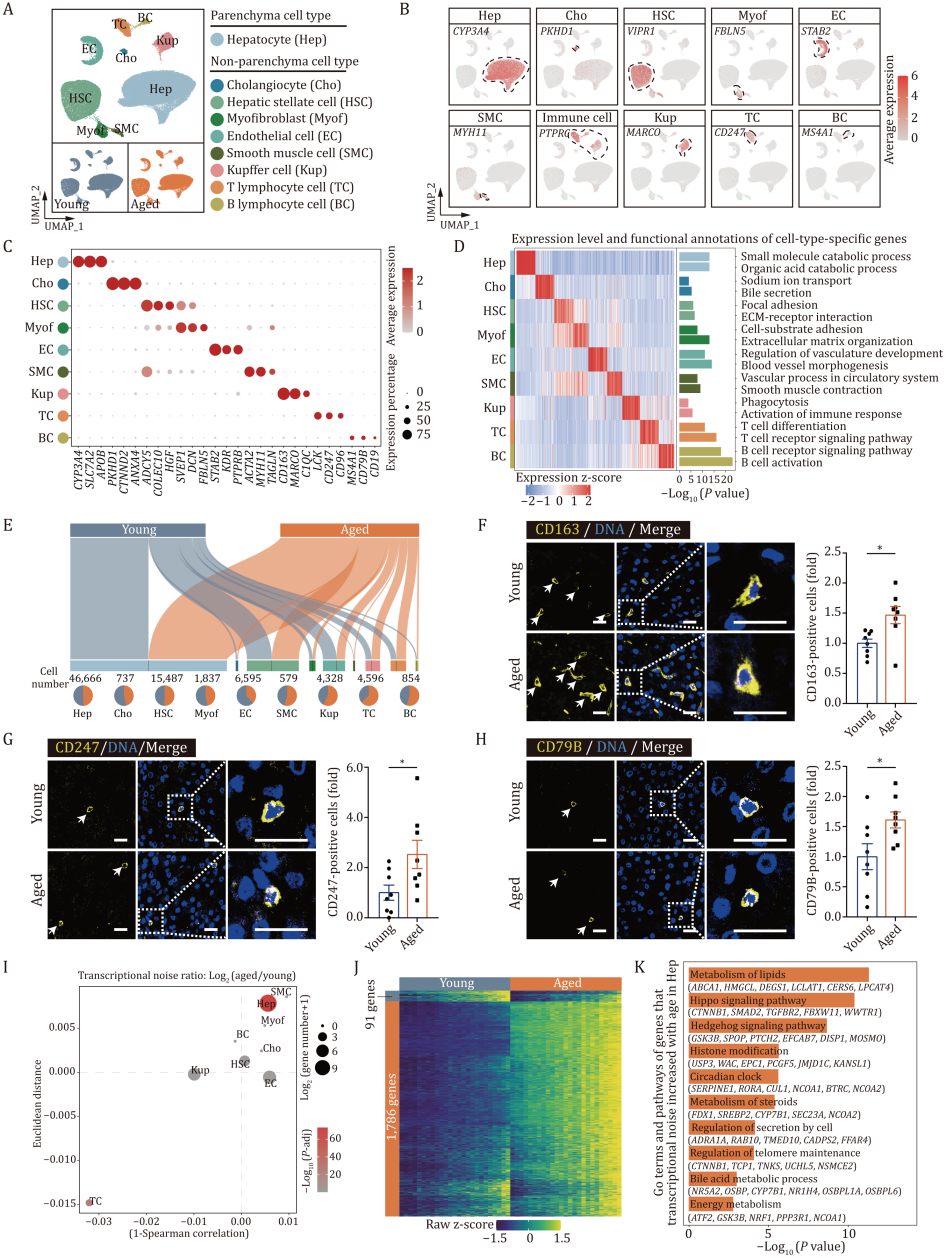
**Single-nucleus transcriptomics identifies major cell types in cynomolgus monkey livers.** (A) UMAP plot showing the distribution of different cell types in liver from young and aged monkeys. (B) Feature plots showing the expression profiles of indicated cell-type-specific marker genes in monkey liver. The color key from gray to red indicates low to high gene expression levels. (C) Dot plot showing the expression level of representative marker genes across cell types. The color key from gray to red presents low to high gene expression levels. The size of dots indicates the percentage of cells with gene expression greater than zero. (D) Heatmap showing the expression profiles of the top 50 cell-type-specific marker genes for each cell type in monkey livers, with corresponding functional annotations on the right. The color key from blue to red represents low to high gene expression levels. (E) Sankey plots showing the cell number of each cell type and the proportion in young and aged monkey livers. The length of the bar indicates the cell number of each cell type, and the number was marked above the pie plot. The pie chart at bottom showing the ratios of each cell type in young and aged monkey livers. (F) Immunofluorescence staining of CD163 in liver tissues from young and aged monkeys. Representative images are shown on the left; quantitative data for the percentage of CD163-positive cells are shown on the right. Scale bars, 20 μm. (G) Immunofluorescence staining of CD247 in liver tissues from young and aged monkeys. Representative images are shown on the left; quantitative data for the percentage of CD247-positive cells are shown on the right. Scale bars, 20 μm. (H) Immunofluorescence staining of CD79B in liver tissues from young and aged monkeys. Representative images are shown on the left; quantitative data for the percentage of CD79B-positive cells are shown on the right. Scale bars, 20 μm. (I) Dot plot showing the log_2_ ratio of transcriptional noise between aged and young samples. The color key from gray to red corresponds to Log_10_ (adjusted *P* value) of transcriptional noise ratio from low to high. The size of dots indicates the number of genes with aging-related transcriptional noise. (J) Heatmap showing the row scaled expression levels of genes with high Pearson’s correlation coefficients (correlation coefficient > 0.6 and FDR < 0.05) between transcriptional noise and expression profiles in hepatocytes from young and aged monkeys. The bins are arranged based on the transcriptional noise ranking in each group. (K) Bar chart showing the enriched GO terms and pathways of genes with increased transcriptional noise during aging in hepatocytes. (F–H) Data are quantified as fold changes and shown as means ± SEM. Young, *n* = 8 monkeys; aged, *n* = 8 monkeys. ^*^P < 0.05.

To comprehensively resolve cell-type-specific responses in liver aging, we isolated and purified 135,757 nuclei from snap-frozen liver samples of young and aged cynomolgus monkeys for snRNA sequencing. After quality control, we used principal component analysis (PCA) dimension reduction followed by graph-based clustering to assigned 81,679 nuclei into 26 clusters, which were visualized by uniform manifold approximation and projection (UMAP) (Fig. S1F–I). Of the nine cell types with distinct cellular transcriptomic signatures, we identified hepatocytes (Hep, 57.13%) and major non-parenchyma cells (NPCs, 42.87%), such as cholangiocytes (Cho, 0.90%), hepatic stellate cells (HSC, 18.96%), endothelial cells (EC, 8.07%), smooth muscle cells (SMC, 0.71%), myofibroblasts (Myof, 2.25%), and Kupffer cells (Kup, 5.30%), T cells (TC, 5.63%), and B cells (BC, 1.05%) (Fig. 3A–C; Table S2). Pathway enrichment analysis of DEGs across cell types showed properties that corresponded to their known biological functions and characteristics (Fig. 3D). Notably, cell identity was confirmed by expression score of cell type-specific marker genes, and we found that the cell identity of all the cell types were compromised during liver aging except T cells (Fig. S1J). Consistent with an earlier study (Michalopoulos, 2017), we did not find any significant influence of aging on predicted hepatocyte cell-cycle activities (Fig. S1K), given that most hepatocytes in homeostasis are expected to be at rest in both young and aged livers. Consistently, the proportions of hepatocytes were not significantly altered during aging (Fig. 3E). In contrast, all proportions of immune cells tended to increase with age (Fig. 3E). When we performed immunofluorescence staining using antibodies for cell markers of Kupffer cells (Buonomo et al., 2022), T cells, and B cells, we verified their increases in the aged liver tissues (Fig. 3F–H), in line with the increased inflammatory response shown at the bulk transcription level (Fig. 2B).

To further analyze aging-related drift in the transcriptome of aged livers, we measured transcriptional noise in different cell types and found the most perturbations in hepatocytes (Fig. 3I). We then identified the genes whose transcriptional fluctuation correlated with increased transcriptional noise in Hep (Fig. 3J). Pathway enrichment analysis showed that dominant genes with fluctuating expression patterns were related to liver homeostasis, such as regulation of telomere maintenance, histone modification, circadian clock, different metabolism-associated pathways (Fig. 3K). Notably, genes involved in important developmental signaling pathways, such as the Hedgehog and Hippo pathways, were the dominant genes underlying aging-related transcriptional noise (Fig. 3K). Both Hippo signaling and Hedgehog signaling are required for liver homeostasis (Omenetti et al., 2011; Gao et al., 2018; Machado and Diehl, 2018; Driskill and Pan, 2021; Russell and Camargo, 2022), and perturbations of downstream signaling pathways in the aged liver therefore reflect perturbed maintenance of metabolic capacity and structural integrity.

### Characterization of cell type-specific transcriptomic changes in liver aging

**Figure 4.**
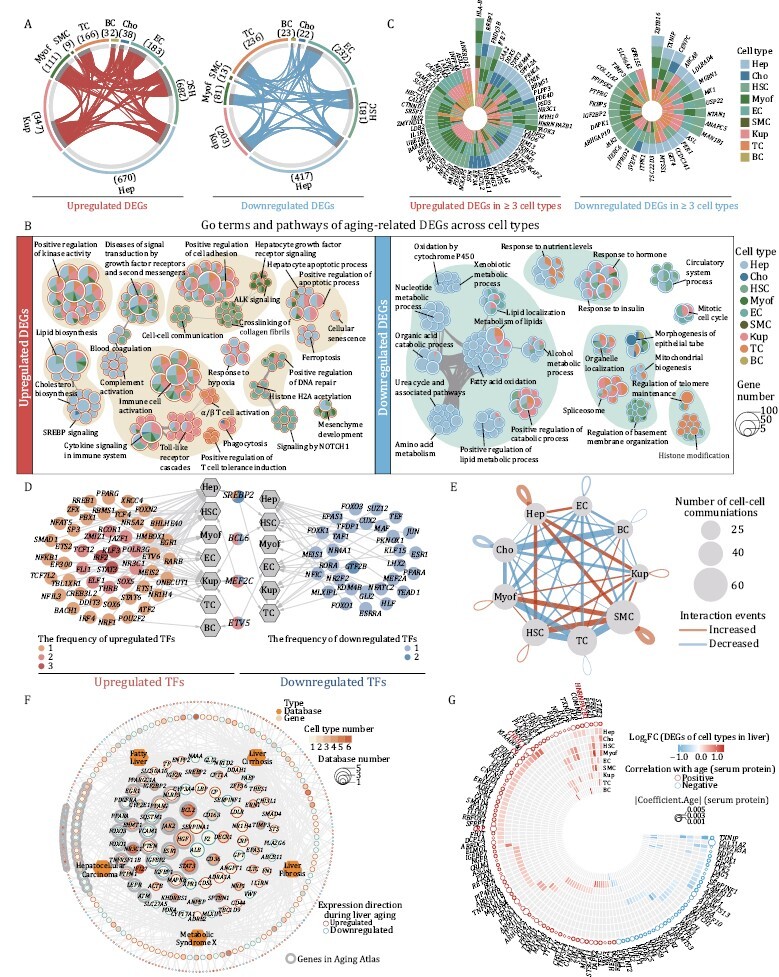
**Characterization of cell-type-specific transcriptomic changes in liver aging.** (A) Circos plots showing aging-related upregulated and downregulated DEGs (adjusted *P* value ≤ 0.05, |Log_2_FC | ≥ 0.25) in different cell types from monkey livers. Each connecting curve represents an upregulated or downregulated DEG shared by two cell types. (B) Network visualizing representative GO terms and pathways of aging-related upregulated (left) and downregulated (right) DEGs in each cell type of monkey liver during aging. The nodes representing GO terms or pathways, the pie plots showing the proportion of gene number that hit the certain GO term or pathway across cell types. Any two nodes with similarity score > 0.3 are connected by a line. (C) Radial plots showing upregulated (left) and downregulated (right) DEGs shared by at least three cell types. (D) Network visualizing the aging-related upregulated (left) and downregulated (right) core regulatory transcription factors (TFs) of each cell type. The hexagon nodes and the circle nodes represent cell types and TFs, respectively. Color keys from light to dark indicate the frequency of TFs from low to high. The pie charts in the middle represent TFs that are both upregulated and downregulated in different cell types. (E) Network showing the changes in ligand–receptor interaction events between different cell types in the aged/young comparison group. Cell–cell communication is indicated by the connected line. The thickness of the lines is positively correlated with the number of ligand–receptor interaction events. Red and blue lines represent increased and decreased interaction events between different cell types. (F) Network visualizing the overlap between aging-related DEGs and liver disease-related genes. The hexagon nodes and the circle nodes represent the types and genes of liver disease database, respectively. The size of gene nodes indicates the frequency of occurrences in liver disease database. The pie-donut charts show the number ratio of aging-associated upregulated (red) and downregulated (blue) genes across cell types. Genes in Aging Atlas database are marked with gray background. (G) Ring heatmap showing the co-upregulated and co-downregulated genes between snRNA-seq and aging-related human plasma proteome dataset. The circles represent the aging-related proteins and the size of circles shows the coefficient of aging-related proteins. The colors of circle (red and blue) represent positive and negative correlation with aging, respectively. The color key of heatmap from blue to red indicates the Log_2_FC of DEGs in snRNA-seq from low to high.

Next, we systemically characterized DEGs associated with liver aging (aging DEGs) in all cell types. Overall, we identified 2,404 aging DEGs (Log_2_FC ≥ 0.25, adjusted *P* value ≤ 0.05) that were presented in at least one cell type in young and aged livers (Fig. 4A; Table S3). The most affected cell types during aging included Hep, Kup, and EC, with aging DEG counts of 1,087, 550, and 415, respectively (Fig. 4A).

When we focused on aging DEGs for each cell type, we found more specific upregulation of genes associated with cholesterol biosynthesis (*HMGCR*, *SQLE*, *SREBP2*, etc.), complement activation (*APCS*, *C5*, *C6*, etc.), cellular senescence (*NFKB1*, *PTEN*, *RAF1*, etc.) and ferroptosis (*CP*, *ACSL4*, *TF*, etc.), and downregulation of xenobiotic metabolic process (*CYP2C18*, *FMO5*, etc.), oxidation by cytochrome P450 (*CYP2E1*, *CYP3A4*, *CYP17A1*, etc.), nucleotide metabolic process (*NAXD*, *OGDHL*, *AFMID*, etc.), and amino acid metabolic process (*ARG2*, *CPS1*, *SDS*, etc.) in aged Hep (Figs. 4B and S2A). Notably, we detected activation of SREBP signaling (*SREBP2*, *ACLY*, *CYP51A1*, etc.) in aged Hep and Cho, and genes associated with phagocytosis were upregulated in both aged Cho and immune cells (Figs. 4B and S2A). There were no apparent specific upregulated terms for aged Cho, but epithelial tube morphogenesis-related genes (*ERBB4*, *ESR1*, etc.) were specifically downregulated in aged Cho (Figs. 4B and S2A), suggesting a potential disruption of the biliary tract structure in aged liver (Banales et al., 2019). In aged HSC, we found that a series of collagen-encoding genes were specifically upregulated, including *COL1A2*, *COL4A1*, *COL4A2*, etc., but that genes related to regulation of basement membrane organization (*LAMA2*, *LAMB1*, *PHLDB1*, etc.), which are critical for maintenance of hepatocyte quiescence (Rohn et al., 2018), were downregulated (Figs. 4B and S2A). Consistently, we noted specific upregulation of genes enriched in ALK signaling in aged Myof (*CLTC*, *RPS6*, *STAT3*, etc.), which is studied extensively in liver fibrosis (Figs. 4B and S2A). In line with the chronic inflammatory phenotypes, genes associated with Toll-like receptor cascades (*CUL1*, *TANK*, *TAB2*, etc.) were activated in Kupffer cells, and positive regulation of T cell tolerance induction genes (*CBLB*, *TGFBR2*, *ITCH*, etc.) were upregulated in TC (Figs. 4B and S2A). “Signaling by NOTCH1” pathway genes (*EP300*, *NCOR1*, etc.) were in general found to be enriched in EC from aged livers, consistent with compensatory and adaptive vascular niche remodeling in aged livers (Figs. 4B and S2A).

Even though expression of aging DEGs varied across different cell types, some common genes were shared across the major cell types. Upregulated DEGs were associated with diseases of signal transduction by growth factor receptors and second messengers (*CAMK2D*, *ESR1*, *SMAD4*, etc.) and cytokine signaling in the immune system (*ANXA3*, *IFNGR1*, *IL6R*, etc.), whereas, common downregulated aging DEGs were enriched in regulation of organelle localization (*ATM*, *ACTN4*, etc.), positive regulation of catabolic process (*APOC2*, *PPARA*, *LRP1*, etc.), and response to hormone (*CPS1*, *CTSB*, *FOXO3*, etc.) (Figs. 4B and S2A). In line with the annotated upregulated pathway, *HLA-B*, a member of the Class I major histocompatibility complex that presents antigens to T cells, was upregulated in seven of the nine cell types (Fig. 4C). In addition, *DDX5*, a key regulator of the IFN response dynamics that is also related to the immune response, was upregulated in four cell types (Fig. 4C). In contrast, *ZBTB16*, a pleiotropic transcription factor associated with regulation of adipogenesis, lipid levels, and insulin sensitivity, was downregulated in most liver cell types (Fig. 4C) (Šeda et al., 2017). To untangle the transcription connectivity between core nodes and targets altered during liver aging, we performed single-cell regulatory network inference and clustering (SCENIC) analysis and predicted core transcription factors regulating aging DEGs across different cell types (Fig. 4D). We identified upregulated regulons for liver aging DEGs, such as *STAT3* and *IRF2*, well-known nodal genes for immune response regulation and tumorigenesis (Guo et al., 2021). We also noticed that *TEAD1*, which plays a key role in the Hippo signaling pathway, was downregulated in aged livers. In addition, four genes were identified as core nodes for liver aging DEGs in a cell-type-dependent manner (Fig. 4D). Among them, *SREBP2*, which plays a crucial role in cholesterol and lipid biosynthesis, was upregulated specifically in Hep but downregulated in HSC and TC (Fig. 4D) (Eberlé et al., 2004; Hillmer et al., 2016; Zhao et al., 2021). Collectively, these data provide insights into the cell-type-specific transcription regulatory network for primate liver aging.

### Aberrant cell–cell communications and expression of disease-related hotspot genes in the aged liver

To understand whether aging leads to an altered microenvironment in primate livers, we constructed a cell–cell interaction network with weighted edges reflecting fold changes in ligand-receptor expression. In all, we identified 211 aged group-specific cell–cell interactions and 253 young group-specific cell–cell interactions, among which Hep appeared to communicate more with HSC, SMC and Kup cells, besides, SMC also exhibited a stronger interaction with HSC in aged liver (Figs. 4E and S2B; Table S4). Top-weighted interactions among these cell types in aged liver were driven mostly by collagens, including *COL4A6*, and TGFβ signals, including *TGFB3*, consistent with reported interactions promoting tissue fibrosis in various disease conditions (Figs. 4E and S2B, S2C) (Branton and Kopp, 1999; Fabregat et al., 2016; Karsdal et al., 2017; Kim et al., 2018). Notably, ligand-receptor pairs that activate Notch signaling and mTOR pathway, that help regulate sugar and fat metabolism (Adams and Jafar-Nejad, 2019; Byles et al., 2021), were decreased in aged HSC and Myof, etc (Fig. S2B and S2C). These identified interactions are consistent with an impaired metabolic function in the aged liver.

Aging is recognized as a major risk factor for chronic diseases, including various liver diseases (Schmucker, 2005). Using the Aging Atlas (2021) and databases comprising hotspot genes for liver diseases or their related complications (Piñero et al., 2021), including fatty liver, liver fibrosis, liver cirrhosis, hepatocellular carcinoma, and metabolic syndrome X, we found that many aging DEGs of liver overlapped with the genes in the above-mentioned databases (Fig. 4F; Tables S5 and S6). Among them, hepatocyte function-related genes, such as *ALB* and *CYP3A4*, as liver-disease risk factors, were downregulated in aged liver (Fig. 4F; Table S5). Whereas the gene *CRP*, which encodes the C-reactive protein, a marker of chronic inflammation (Gorabi et al., 2022) that has been associated with liver diseases, was upregulated in aged liver (Fig. 4F; Table S5). Moreover, α1-antitrypsin (encoded by *SERPINA1*), an inhibitor of neutrophil elastase, whose gene variants are associated with severity of fatty liver disease, liver fibrosis and cirrhosis (Basyte-Bacevice et al., 2019; Semmler et al., 2021), was also upregulated in aged liver (Fig. 4F; Table S5).

Since the liver is a unique organ that holds more than 10% of total human blood supply and secretes a variety of proteins into the blood to support a wide range of physiological functions (Stefan and Häring, 2013; Jensen-Cody and Potthoff, 2021), we integrated the analysis of our snRNA-seq dataset with a previously published serum proteomic dataset across the lifespan (Lehallier et al., 2019) and identified a total of 112 overlapped factors between aging DEGs and aging-associated differentially expressed serum proteins (Fig. 4G; Table S7). Among them, *CHI3L1*, a previously reported noninvasive biomarker for stage diagnosis of hepatic fibrosis, was specifically upregulated in the aged Hep and serum (Wang et al., 2020a). Similarly, hnRNPK, which is encoded by *HNRNPA2B1* and used as a biomarker in early HCC, was also upregulated in most of the cell types of aged liver (Fig. 4G; Table S7). Based on our analysis, the complement protein *C9* was also highlighted as being increased in the aged liver and plasma (Fig. 4G; Table S7). Interestingly, we also found that *CRP* was upregulated in both aged Hep and plasma (Fig. 4G; Table S7). Further pathway enrichment analysis for overlapping factors revealed that the upregulated ones were functionally enriched in interleukin and TGFβ signaling (Fig. S3A). In contrast, downregulated genes were enriched for factors required for blood vessel development and response to hormone (Fig. S3A). Taken together, our results delineate how cell-type specific gene expression and abnormal cell–cell interactions intersect in aged liver.

### Zonation-specific transcriptional alterations in aged cynomolgus monkey livers

**Figure 5.**
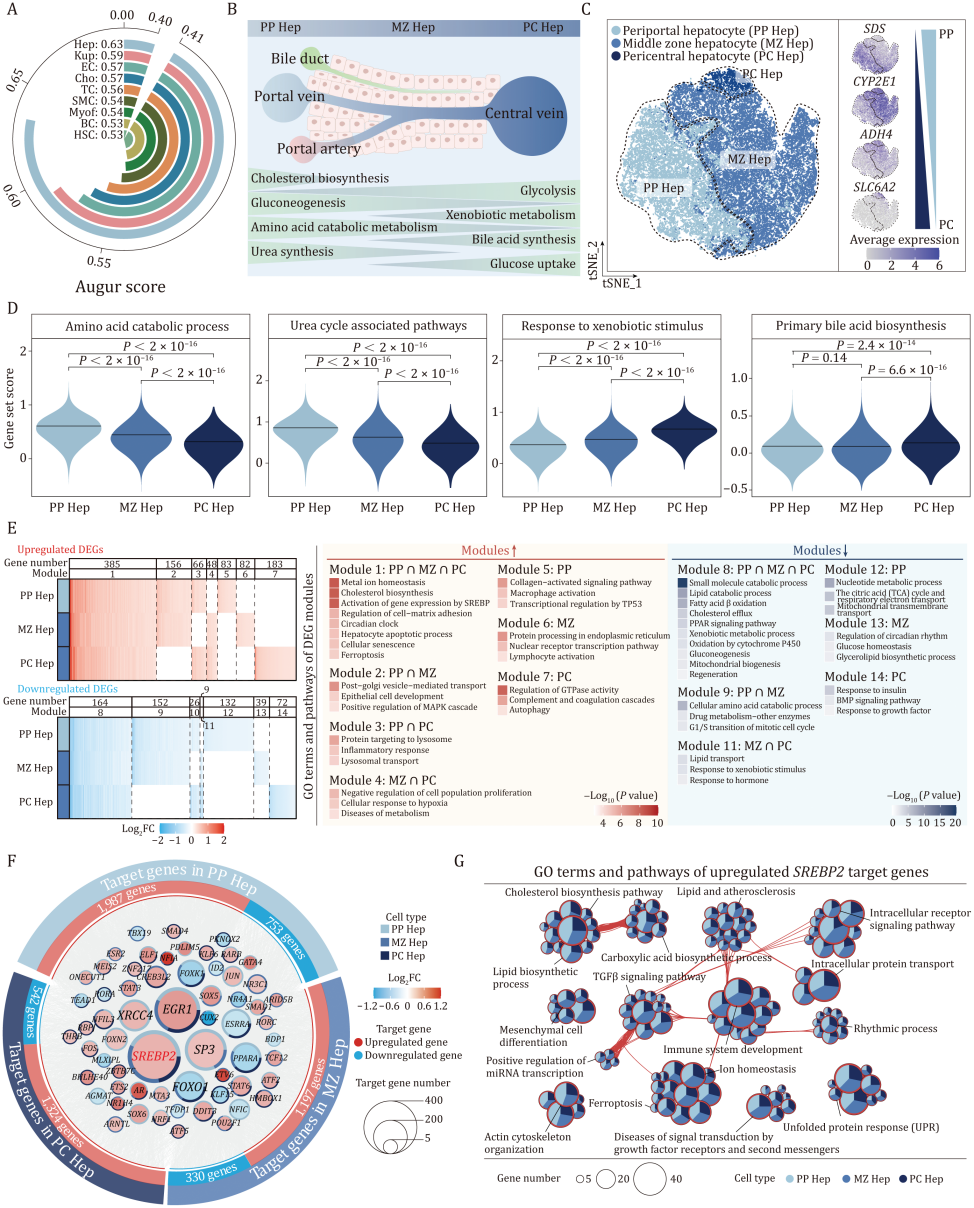
**Zonation-specific transcriptional alterations in aged cynomolgus monkey livers.** (A) Arc plot showing prioritization of the cell types responsive to aging. (B) Schematic diagram showing the structure and functions of liver lobules. (C) tSNE plot showing the distribution of hepatocyte subtypes (left) and the expression levels for cell-type-specific marker genes of each subtype (right). The color key from gray to blue indicates low to high gene expression levels. (D) Violin plots showing the score of gene sets related to zonation-specific functions across three hepatocyte subtypes. (E) Heatmap (left) showing the aging-related DEGs across three hepatocyte subtypes. DEGs are classified into 14 modules according to the overlap among three subtypes. Heatmap (right) showing the GO terms and pathways of modules. (F) Network visualizing aging-related upregulated and downregulated core regulatory TFs (nodes) and their target genes (points) among three hepatocyte subtypes, with TFs arranging in the middle and target genes arranging on the outer ring. The size of the node indicates the number of target genes regulated by the TFs. The colors of node represent the average Log_2_FC of TFs during aging in different hepatocyte subtypes. Red and blue points represent upregulated and downregulated target genes during aging respectively. The pie-donut chart shows the number ratio of target genes regulated by the TFs across allhepatocyte subtypes. (G) Network visualizing the enriched GO terms and pathways of upregulated *SREBP2* target genes among three hepatocyte subtypes. The nodes representing GO terms and pathways, the pie plots showing the proportion of gene number that hit the certain term or pathway across three hepatocyte subtypes. Any two terms with similarity score > 0.3 are connected by a line.

Through the use of Augur analysis, a bioinformatics toolkit that enables users to track evolution from sequence and serological data (Skinnider et al., 2021), we identified that Hep was the cell type most responsive to aging in the single-nucleus data (Fig. 5A). We also noticed that most of the aging DEGs detected at bulk RNA-seq level were in Heps, consistent with important implications for liver aging (Fig. S3B and S3C). Thus, these results highlighted that, hepatocytes, the main functional cells in liver, were highly vulnerable to aging. Hepatocytes line different zonation along the portal–central axis in liver lobules, defined by differences in gene expression, enzyme activity, and metabolic functions (Fig. 5B) (Ben-Moshe and Itzkovitz, 2019). To precisely unravel such zonation-specific aging-related changes in hepatocytes, we further characterized the transcriptomic data for the hepatocyte population using defined maker genes associated with liver zonations (Torre et al., 2010; Ben-Moshe and Itzkovitz, 2019; Annunziato and Tchorz, 2021; Paris and Henderson, 2022).

We identified three cell clusters in Hep, of which cluster 1 was assigned as periportal hepatocytes (PP Hep), which specifically express *SDS*; cluster 2 was designated as pericentral hepatocytes (PC Hep), which highly express *ADH4* and *SLC6A2*; and cluster 3 was designated as middle zone hepatocytes (MZ Hep) (Figs. 5C and S3D; Table S8). There were relatively few highly expressed marker genes in MZ Hep, but instead we detected a transcriptional gradient from PP Hep to PC Hep (Figs. 5C and S3D). The three hepatocyte subtypes had their own well-recognized zonation-specific metabolic properties; for example, genes related to amino acid catabolism and urea cycle associated pathways were highly expressed in PP Hep, whereas genes related to response to xenobiotic stimulus and primary bile acid biosynthesis were more strongly expressed in PC Hep (Fig. 5D; Table S9) (Braeuning et al., 2006; Schleicher et al., 2015; Kietzmann, 2017). Overall, the molecular traits of PP Hep, and PC Hep were consistent with their reported physiological functions in the metabolism of amino acids and xenobiotics, respectively.

Next, we further characterized whether the aging-associated changes in hepatocytes depended on their partition location. All Hep subtypes showed a comparable number of aging DEGs (Fig. 5E; Table S10); accordingly, Augur analysis (Skinnider et al., 2021) showed minor differences across these three hepatocyte subtypes (Fig. S3E). Although more than half of aging DEGs were shared in at least two zonation subtypes, each Hep subtype had its own specific aging signature (Fig. 5E; Table S10). Aging DEGs of PP Hep included upregulation in the collagen-activated signaling pathway and suppression in the tricarboxylic acid (TCA) cycle and respiratory electron transport, while upregulation of genes involved in protein processing in the endoplasmic reticulum and downregulation of regulatory members of circadian rhyme were features in MZ Hep (Fig. 5E; Table S10). On the other hand, aged PC Hep showed enriched gene expression related to complement and coagulation cascades, but less in genes related to response to insulin (Fig. 5E; Table S10). Although metabolic pathway-related genes were commonly downregulated in all three hepatocyte subtypes during aging (Fig. 5E), each subtype of hepatocytes had a well-maintained gene expression pattern associated with region-specific metabolic features; genes related to amino acid catabolism and urea cycle associated metabolic functions were relatively highly expressed in PP Hep, and genes responsive to xenobiotic stimulus and primary bile acid biosynthesis were highly expressed in PC Hep (Fig. S3F), suggesting that even though hepatic aging was accompanied by downregulation of metabolism-related genes, their zonation identity was preserved. Taken together, we here partitioned hepatocytes into three zonation subtypes based on their transcriptomic features and identified their common and specific aging-related gene expression changes.

To discover key regulons for the gene expression changes in aged Hep subtypes, we used SCENIC to analyze core TFs for all the Hep subtypes or each subtype within them. Each subtype was predicted to have its own regulatory TFs, such as *SMAD4* for upregulated aging DEGs in PC Hep, *NR3C1* for upregulated aging DEGs in MZ Hep, and *XRCC4* for PP Heps (Fig. 5F). Notably, we identified 14 upregulated and 7 downregulated TFs that were involved in gene regulation of all three Hep subtypes (Fig. 5F). Of these, *SREBP2* functioned as the upstream hub most broadly regulating aging DEGs (524) in all three Hep subtypes (Fig. 5F). Pathway enrichment analysis of *SREBP2*-upregulated target DEGs revealed potential roles in cholesterol and lipid biosynthetic processes, unfolded protein response (UPR), TGFβ signaling and mesenchyme development, and immune system development, which encompass nearly all major changes associated with liver aging (Figs. 5G and S3G), suggesting that *SREBP2* is a core regulator of liver aging. In conclusion, our analysis points to an important role for *SREBP2* and its downstream target genes in Hep aging, and therefore, targeting this gene and related signaling pathways holds potential for interventions in liver aging.

### SREBP2 mediates senescence and metabolic dysfunction in hepatocytes

**Figure 6.**
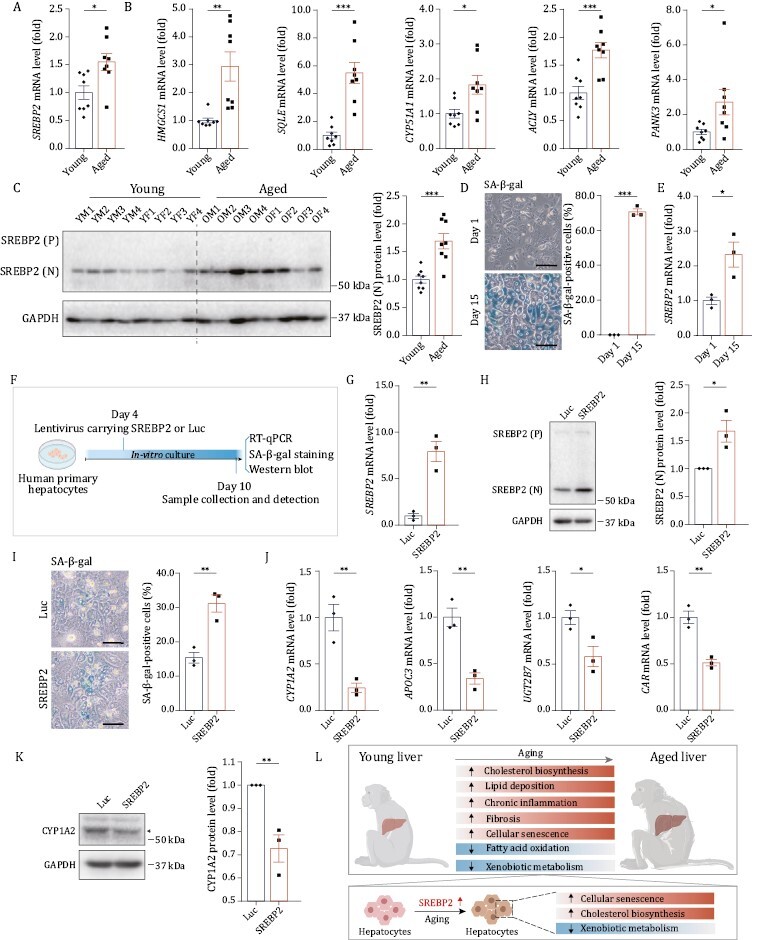
**SREBP2 mediates senescence and metabolic dysfunction in hepatocytes.** (A) RT-qPCR analysis of *SREBP2* mRNA level in liver tissues from young and aged monkeys. (B) RT-qPCR analysis for mRNA levels of classical target genes of *SREBP2* in liver tissues from young and aged monkeys. (C) Western blot for protein level of SREBP2 (N) in liver tissues from young and aged monkeys. SREBP2 (P), precursor SREBP2 protein; SREBP2 (N), nuclear SREBP2 protein. Representative images are shown on the left; quantitative data for the SREBP2 (N) protein level are shown on the right. (D) SA-β-gal staining was performed on human primary hepatocytes cultured *in vitro* on Day 1 and Day 15 respectively. Representative images are shown on the left; quantitative data for the percentage of SA-β-gal positive cells are shown on the right. Scale bars, 100 μm. (E) RT-qPCR analysis of *SREBP2* mRNA level in human primary hepatocytes cultured *in vitro* on Day 1 and Day 15, respectively. (F) Schematic of experiments in human primary hepatocytes transduced with lentiviruses expressing SREBP2 or luciferase (Luc, used as control). Created with BioRender.com. (G) RT-qPCR analysis of *SREBP2* mRNA level in human primary hepatocytes transduced with lentiviruses expressing SREBP2 or Luc. (H) Western blot for protein level of SREBP2 (N) in human primary hepatocytes transduced with lentiviruses expressing SREBP2 or Luc. SREBP2 (P), precursor SREBP2 protein; SREBP2 (N), nuclear SREBP2 protein. Representative images are shown on the left; quantitative data for the protein level of SREBP2 (N) are shown on the right. (I) SA-β-gal staining in human primary hepatocytes transduced with lentiviruses expressing SREBP2 or Luc. Representative images are shown on the left; quantitative data for the percentage of SA-β-gal-positive cells are shown on the right. Scale bars, 100 μm. (J) RT-qPCR analysis for mRNA levels of liver function-related genes in human primary hepatocytes transduced with lentiviruses expressing SREBP2 or Luc. (K) Western blot of CYP1A2 protein level in human primary hepatocytes transduced with lentiviruses expressing SREBP2 or Luc. Representative images are shown on the left; quantitative data for the protein level of CYP1A2 are shown on the right. (L) Schematic diagram showing the signatures of primate liver aging. Created with BioRender.com. Data are quantified as fold changes (excluding D and I) and shown as means ± SEM. For (A–C), young, *n* = 8 monkeys; aged, *n* = 8 monkeys. For (D, E and G–K), *n* = 3 biological replicates. ^*^P < 0.05, ^**^P < 0.01, ^***^P < 0.001

In line with the snRNA-seq analysis, we validated that transcript levels of *SREBP2* and several of its downstream target genes (*HMGCS1*, *SQLE*, *CYP51A1*, *ACLY*, *PANK3*) were all elevated in aged livers (Fig. 6A and 6B). Accordingly, based on western blotting, elevated levels of nuclear SREBP2 protein, the activated form of SREBP2, were detected in aged livers (Fig. 6C). Taken together, these data indicate that activation of SREBP2 and its downstream signaling may be a main feature of hepatic aging.

To further verify the involvement of SREBP2 in hepatocyte aging, we established an *in vitro* senescence model of human primary hepatocytes in which the proportion of senescence-associated β-galactosidase (SA-β-gal) positive cells was gradually increased during prolonged *in vitro* culture (Fig. 6D). Consistent with the *in vivo* observation in aged hepatocytes, the transcript level of *SREBP2* was also upregulated in senescent hepatocyte *in vitro* (Fig. 6E). Next, we investigated the functional role of SREBP2 by transducing human primary hepatocytes with lentiviruses expressing SREBP2 (Fig. 6F). Quantitative RT-PCR analysis and western blot assay confirmed that the expression of SREBP2 was increased after lentiviral transduction (Fig. 6G and 6H). In line with the above-mentioned results, SREBP2 target genes that are related to cholesterol biosynthesis (*HMGCS1*, *SQLE*, *CYP51A1*, *ACLY*) were also upregulated in human hepatocytes with ectopic expression of SREBP2 (Fig. S3H). Compared to the control group, *SREBP2-*transduced hepatocytes exhibited accelerated senescence, as evidenced by increased SA-β-gal positive cells (Fig. 6I), and compromised expression of CYP family genes (*CYP1A2*), lipid and cholesterol metabolism genes (*APOC3*), and xenobiotic metabolism genes (*UGT2B7*, *CAR*) (Fig. 6J and 6K). Altogether, these results support a mechanistic role for SREBP2 in driving senescence and functional decline in human hepatocytes during aging and are consistent with our observations in monkey livers (Fig. 6L).

## Discussion

In this study, we presented a transcriptomic atlas of young and aged livers from healthy cynomolgus monkeys at a single-cell resolution, and interrogated cell type-specific gene expression changes and the microenvironmental circuitry underling the hepatic aging phenotypes. From the derived comprehensive landscape for liver aging, we revealed that all three liver zonations were highly sensitive to aging, and identified SREBP2 as a hub regulon whose activation triggered hepatocyte senescence and dysfunction. Our data shed light on the molecular basis of liver aging, which might guide the development of evaluation and intervention strategies for liver aging to benefit a wide range of people.

Hepatocytes in the liver, even in adulthood, possess strong regenerative capacity (Gadd et al., 2020; Campana et al., 2021; He et al., 2021; Ben-Moshe et al., 2022), and as a result, the liver is generally considered to be a relatively “aging-resistant” organ. In our study, we found that the liver underwent marked changes during aging, both at the level of tissue structure and function as well as in cell proportions and molecular changes. At the tissue level, accumulation of senescent cells, increased chronic inflammation, impaired metabolism and detoxification, and aggravated fibrosis were hallmarks of the aged liver. Furthermore, at the transcriptional level, expression of cell-identity genes associated with certain cell types, especially cholangiocytes, EC and SMC, was decreased. And transcription fluctuation in hepatocytes was dramatically increased. Given the above-mentioned changes, hepatocytes appeared to be the cell type most affected by aging. Thus, despite their intrinsic regenerative capacity, hepatocytes gradually lose cellular homeostasis and enter senescence as the body ages, which in turn compromises detoxification and liver metabolism functions. Because the liver maintains the metabolic and detoxification homeostasis of the entire body, aging-associated impaired liver function is closely linked with whole-body aging and may even be the main driving force of aging. Indeed, in a recent study in mice, delaying the senescence of hepatocytes largely improved liver function, thereby slowing down the aging of the whole body and extending the healthy lifespan of mice (Wang et al., 2021a). These functional observations, in combination with our work, support the idea that hepatocyte senescence drives liver aging and even systematic aging, and therefore, rejuvenation of the senescent hepatocytes may be a potent intervention strategy in both liver and systemic aging.

The single-cell transcriptomic approach is changing our understanding of the cellular and molecular diversity of complex tissues in both health and disease conditions (Potter, 2018; Stark et al., 2019; Saviano et al., 2020; Leng and Pawelec, 2022; Fang et al., 2022). With the advent of these powerful techniques, previous seminal work studying liver physiology and pathobiology has been completed (Ramachandran et al., 2020). However, due to ethical issues and clinical limitations, primate liver studies, especially aging studies, using single-cell techniques have remained outstanding. MacParland et al. (2018) published scRNA-seq profiling for liver grafts from five neurologically deceased donors and first identified hepatocyte subtypes correlated with human liver zonation. Next, Aizarani et al. (2019) performed scRNA-seq analysis of livers from patients who underwent liver resection due to cancer metastasis and reported limited evolutionary conservation on gene expression between humans and mice. Here, we analyzed cellular and spatial heterogeneity during primate liver aging. Using single-nucleus RNA-seq, which recapitulates cell proportions better than scRNA-seq, and is superior for an unbiased dissection of all cell types present in the liver (Bakken et al., 2018; He et al., 2020), we interrogated the contribution of different cell types to primate liver aging. Our work provided important insights that will help reshape our understanding of aging-associated changes occurring in hepatocyte subtypes, and which might become pivotal for the identification of cell-specific intervention targets in liver aging.

Senescent cells accumulate in various tissues with age, including the liver (O’Hara and La Russo, 2017; Ogrodnik et al., 2017; Ma et al., 2020). These senescent cells arise in the liver under pathological conditions that adversely affect liver function and tissue regeneration (Aravinthan et al., 2013; He and Sharpless, 2017; Bird et al., 2018; Sun et al., 2022). Based on mouse models, oxidative stress was suggested as a link to DNA damage that caused hepatocyte senescence in chronically injured livers (Xiao et al., 2018). In addition, a recent study reported that suppression of autophagic activity via mTORC1/TFEB signaling exacerbate hepatocyte senescence via growth differentiation factor 11 (GDF11) (Sun et al., 2022). Moreover, the accumulation of lipid droplets in hepatocytes is known from earlier work to promote telomere shortening and DNA damage, which may induce senescence in hepatocytes (Campisi and di Fagagna, 2007; Huda et al., 2019). These aforementioned dysregulated molecular events in hepatocytes have mainly been reported in injury or disease conditions, but in our study, we observed the above-mentioned oxidative stress, compromised mTOR signaling, and the accumulation of lipid droplets in the livers of aged monkeys.

It has been widely reported that SREBPs promote the synthesis of fatty acids, triglycerides and cholesterol to support lipid metabolism (Eberlé et al., 2004). In our study, SREBP2, a member of the SREBP family that primarily regulates genes involved in cellular cholesterol homeostasis (Shimano, 2009; Soyal et al., 2015), was found to be highly expressed in the aged liver. The activated SREBP2 is reported to increase hepatic cholesterol levels by promoting cholesterol influx (Maxfield and Tabas, 2005; Van Rooyen and Farrell, 2011; Shao and Espenshade, 2012), thereby leading to cytoplasmic lipid droplets (Horton et al., 2002; Maxfield and Tabas, 2005). However, the regulatory role of SREBP2 in cellular senescence, especially hepatocyte senescence, is barely recognized. A previous study linked the longevity gene SIRT6 to SREBP through deacetylation of AMPK and subsequent phosphorylation and inhibition of SREBP, suggesting a potential role for SREBP linking energy homeostasis and aging (Elhanati et al., 2013). More recently, mature SREBP2 was recognized as a transcription factor activating the expression of a series of inflammatory cytokines and profibrotic factors, including IL1β and collagen V1 (Dorotea et al., 2020; Lee et al., 2020), which coincided with aggravated inflammation and fibrosis in aged monkey livers. In our study, we identified SREBP2 as an upstream hub regulon for most of the transcriptomic changes in all zonation subtypes of hepatocytes from aged monkeys. Most importantly, overexpression of SREBP2 in human primary hepatocytes recapitulated aging phenotypes observed in aged monkey livers, as manifested by induction of cellular senescence and decreased expression of genes related to metabolism and detoxification function. Our work demonstrated, the important driving role of SREBP2 in liver aging, linking its activity to increased cellular senescence and impaired liver functions in the aged liver, thereby highlighting its potential as a key target for aging interventions.

Our study brings to light the little-understood mechanisms of liver aging in primates. Because many tissues and organs depend closely on liver function, liver aging has both direct and indirect impacts, making it a crucial area for understanding systemic aging. Therefore, our findings are of importance, as our comprehensive study of liver aging helps us uncover key cellular and molecular targets with potential to serve as biomarkers to predict and monitor liver or body aging. Conversely, we also identified SREBP2 as an important driving force of hepatocyte senescence, whose manipulation would be expected to slow the aging process and delay the onset of aging-related diseases.

## Materials and methods

### Animals

The cynomolgus monkeys used in this study are eight young monkeys (4–6 years old) and eight aged monkeys (18–21 years old), both groups of which have been approved by the Ethical Review Committee of the Institute of Zoology, Chinese Academy of Sciences (Zhang et al., 2020). They were raised at facility in Xieerxin Biology Resource (a certified primate research center in Beijing) at 25°C with a 12-h light and dark cycle. Before the start of the experiment, it was confirmed that all animals used had no clinical disease, experimental experience, or history of pregnancy. The detailed information of the animals used is shown in Fig. S1A.

### Tissue sampling

The cynomolgus monkeys were fully anesthetized and perfused with phosphate buffer. Liver tissue was then intactly isolated according to histological anatomy with attached fat tissue being removed. Then the liver tissue was cut to nearly uniform size and stored in liquid nitrogen for subsequent sequencing analysis, as well as other biochemical and molecular analyses.

### Hematoxylin–eosin staining

Hematoxylin–eosin staining (HE staining) was performed as described earlier (Zou et al., 2021). The sections were dehydrated in gradient alcohol (70%–100%) and soaked in 4% paraformaldehyde (PFA). The sections were then embedded in paraffin and sectioned at a thickness of 5 μm using a rotary microtome, sections placed on a glass slide, allowed to dry at 56°C for 2 h, and then stored at room temperature (RT) for later use. During staining, the sections were deparaffinized in xylene, rehydrated in gradient alcohol (100%, 100%, 95%, 80%, 75%), and rinsed briefly in distilled water, and then incubated in hematoxylin solution until the required degree of staining was achieved, assessed under microscope (Servicebio, China), and then rinsed in running water to remove excess hematoxylin solution. Slices were then differentiated in 1% acidified alcohol for 1 s and rinsed in running water for 1 min. Finally, the sections were stained with eosin until achieving the desired pink color, dehydrated in gradient alcohol and xylene, and mounted with cytoseal-60 (Stephens Scientific). Images were taken by PerkinElmer Vectro Polaris and the inflammation area was quantified using Image J.

### Masson-trichrome staining

Masson-trichrome staining was performed as previously described (Lei et al., 2021). Paraffin-embedded sections with a 5 μm thickness were deparaffinized in xylene and rehydrated in gradient ethanol (100%, 100%, 95%, 80%, 75%). Sections were then rinsed with distilled water and stained with potassium dichromate solution at 60°C for 1 h. After rinsing in running water for 5–10 min, sections were stained with iron hematoxylin working solution for 10 min, and rinsed again in warm running water for 10 min. Then, sections were stained with Ponceau-acid fuchsin solution for 5–10 min and rinsed in distilled water, differentiated in the phosphomolybdic-phosphotungstic acid solution for 10–15 min or until the red color on the tissue disappeared. Rinsing was no longer necessary at this time, and sections were stained directly in aniline blue solution for 5–10 min, then rinsed briefly in distilled water and differentiated in 1% acetic acid solution for 2–5 min. Finally, after washing in distilled water several times, sections were quickly dehydrated with 95% ethanol and absolute ethanol, cleared in xylene, and mounted with a resin mounting agent. Images were taken by PerkinElmer Vectro Polaris and the fibrotic area of liver parenchyma excluding vascular area was quantified using Image Pro plus.

### Sudan Black B staining

Sudan Black B staining (SBB staining) was performed using a previously published protocol (Georgakopoulou et al., 2013). In short, OCT-embedded and snap-frozen tissues were cryosectioned at a thickness of 10 μm with a Leica CM3050S cryomicrotome. Frozen sections were taken out of the refrigerator at −80°C, placed at RT for a few minutes and fixed in 1% (*w*/*v*) formaldehyde/PBS for 5 min at RT, and then rinsed gently with distilled water three times, incubated in 50% ethanol and 70% ethanol for 5 min each in turn. Next, frozen sections were stained in Sudan Black B solution (0.7 g SBB in 100 mL 70% ethanol) for 5 min at RT, and then rinsed quickly in 75% ethanol to remove excess staining solution, rinsed with distilled water three times. Finally, sections were left to stain in nuclear fast red solution for 3 min, washed in distilled water and mounted with glycerol. Images were taken with Olympus CKX41 microscope imaging system, and Image Pro Plus was used to quantify the Sudan Black B positive area.

### Immunohistochemistry staining

Immunohistochemistry staining was performed as previously described (Wang et al., 2020b). Paraffin-embedded sections were first deparaffinized in xylene and then hydrated with gradient alcohol (100%, 100%, 90%, 80%, 70%, 50%). After rinsing in distilled water for a while, slices were microwave-heated in sodium citrate buffer (pH 6.0) three times for antigen retrieval, each for 4 min. After cooling to RT, sections were permeabilized with 0.4% Triton X-100 in PBS for 1 h and incubated with 3% H_2_O_2_ at RT for 20 min to inactivate endogenous peroxidase. Next, sections were blocked with 10% donkey serum for 1 h at RT and incubated with the primary antibody overnight at 4°C. On the second day, sections were incubated with HRP-conjugated secondary antibody for 1 h at RT. Next, sections were colorimetrically detected using DAB and counterstained with hematoxylin, followed by dehydration in a series of graded alcohols (50%, 70%, 80%, 90%, 100%, and 100%) and transfered in xylene before being mounted in the neutral resinous mounting medium. Images were captured by PerkinElmer Vectro Polaris or Leica Aperio VESA8, and statistically quantified of the percentage of positive cells using Image J. The antibodies used for immunohistochemistry staining in this study are listed as follows: anti-P21 (Cell Signaling Technology, 2947S, 1:100), anti-S100A8 (Abcam, ab180735, 1:300), anti-S100A9 (Abcam, ab92507, 1:300), anti-myeloperoxidase (MPO) (Abcam, ab9535, 1:200).

### Immunofluorescence staining

Immunofluorescence staining was performed as previously described (Wang et al., 2020b; Ma et al., 2021). Paraffin-embedded sections were first deparaffinized in xylene and then hydrated with gradient alcohol (100%, 100%, 90%, 80%, 70%, 50%). After rinsing in distilled water for a while, the slices were microwave-heated in sodium citrate buffer (pH 6.0) three times for antigen retrieval, each for 4 min. After cooling to RT, the sections were rinsed in PBS three times, 5 min each time, and permeabilized with 0.4% Triton X-100 in PBS for 1 h. Sections were rinsed again three times in PBS and then blocked with blocking buffer (10% donkey serum in PBS), incubated for 1 h at RT. Then sections were incubated with the primary antibody overnight at 4°C, followed by incubation with fluorescence-labeled secondary antibodies and Hoechst 33342 (Thermo Fisher Scientific) for 1 h at RT in the dark. Finally, sections were mounted in VECTERSHIELD^®^ anti-fading mounting medium (Vector Laboratories, h-1000), and images were obtained using a confocal laser scanning microscope (Zeiss LSM900). For most of immunofluorescence staining in this study, the results were quantified by calculating the percentage of positive cells using Image J. The result of immunofluorescence staining for H3K9me3 performed with quantification of the immunofluorescence intensity for each cell using Image J.

The antibodies used for immunofluorescence staining in this study are as follows: anti-H3K9me3 (Abcam, ab8898, 1:400), anti-TNFα (Abcam, ab1793, 1:100), anti-IL6 (Abcam, ab6672, 1:100), anti-IL1β (Santa Cruz, sc-52012, 1:100), anti-CD68 (Abcam, ab955, 1:300), anti-CD45 (Abcam, ab10558, 1:300), anti-MMP9 (Abcam, ab38898, 1:100), anti-CD163 (Abcam, ab182422, 1:200), anti-CD79B (Cell Signaling Technology, 96024S, 1:200), anti-CD247 (ABclonal, A2058, 1:200). Secondary antibodies used were the following: donkey anti-mouse-AF488 (Thermo Fisher, A21202, 1:500), donkey anti-mouse-AF568 (Thermo Fisher, A10037, 1:500), donkey anti-rabbit-AF488 (Thermo Fisher, A21206, 1:500), donkey anti-rabbit-AF568 (Thermo Fisher, A10042, 1:500).

### SPiDER-βGal staining

Senescence-associated β-galactosidase (SA-β-gal) staining was performed according to the instructions provided by the manufacturer (DojindoMolecular Technologies, Inc., Kumamoto, Japan). First, OCT-embedded liver tissues were cryosected at a thickness of 10 μm using a Leica CM3050S cryosectioner. Then sections were fixed in 4% paraformaldehyde for 20 min at RT and washed in PBS three times. SPiDER-βGal staining working solution was diluted to 20 μmol/L (Dojindo Molecular Technologies, SG03, 1:2,000) with McIlvaine buffer (pH 6.0) and sections were incubated with this working solution overnight at 4°C. Sections were then counterstained with Hoechst33342 (Thermo Fisher, H3570, 1:1000) at RT, washed with PBS three times, and mounted in VECTERSHIELD^®^ Anti-Fade Mounting Medium (Neobioscience, H-1000). Image acquisition was performed using a Zeiss LSM900 confocal system, and data analysis of the percentage of SPiDER-βGal positive cells was performed using image J.

### Oil Red O staining

Oil Red O staining was performed using a previously published protocol (Huang et al., 2022). In short, OCT-embedded and snap-frozen tissues were cryosectioned at a thickness of 10 μm with a Leica CM3050S cryomicrotome. Frozen sections were taken out of the refrigerator at −80°C, placed at RT for a few minutes and soaked in the PBS solution for 5 min. After filtering Oil Red O staining solution (Sigma-Aldrich) through a 100 μm filter, frozen sections were stained in 60% Oil Red O solution for 8–10 min. To avoid background staining, wash the slides with 70% ethanol for a moment, and then rinsed with running tap water and counterstained with hematoxylin. Images were taken with Olympus CKX41 microscope imaging system, and Image Pro plus was used to quantify the oil red O positive area.

### TUNEL staining

In brief, we used the One Step TUNEL Apoptosis Assay Kit (Beyotime, C1088) according to the instruction provided by the manufacturer. Paraffin-embedded sections were deparaffinized in xylene and rehydrated in gradient ethanol (100%, 100%, 95%, 80%, 75%). Proteinase K was diluted with 10 mmol/L Tris-HCl (1:1000), then sections were incubated at RT for 30 min and rinsed with PBS. Next, the sections were stained with TUNEL working solution at 37°C for 1 h, followed by incubation with Hoechst 33342 (Thermo Fisher, H3570, 1:1000) to visualize the nucleus. Finally, the sections were mounted in VECTERSHIELD^®^ anti-fading mounting medium (Vector Laboratories, h-1000), and images were obtained using Zeiss LSM900 confocal system. Image J was used to quantify the percentage of TUNEL-positive cells.

### RNA isolation and RT-qPCR

RNA isolation from cultured cells and frozen tissues in liquid nitrogen was performed as previously described (Wu et al., 2020). The total RNA was extracted with TRIzol (Life Technologies, 15596018). 2 μg of total RNA was taken as a reverse transcription template, and GoScript™ Reverse Transcription System (Promega) was used to reverse transcription to obtain cDNA. RT-qPCR was performed on the CFX384 real-time PCR system (Bio-Rad) using iTaq Universal SYBR Green SuperMix (Bio-Rad). After normalizing the relative mRNA expression level of each detected gene to *GAPDH* expression, it was calculated using the ΔΔCq method. At least three independent samples for each RT-qPCR detection experiment. Analyze the difference between the two groups by independent sample *t*-test. The RT-qPCR primers used in this study were listed in Table S11.

### Western blot analysis

Western blot analysis was performed as described previously (Ma et al., 2022). Both tissues and cells were lysed in RIPA buffer (Beyotime, P0013B) that had been supplemented with protease inhibitors (Roche, 4693159001). After sufficient lysis, the mixture was centrifuged. The supernatant was left for quantification with a BCA kit (Dingguo biotechnology, BCA02). SDS-PAGE electrophoresis and semi-dry membrane transfer were then performed sequentially. PVDF membranes (Merck Millpore) were blocked with 5% skim milk for 1 h at RT and then incubated with primary antibody overnight at 4°C. The next day, PVDF membranes were washed with TBST buffer and incubated HRP-conjugated secondary antibodies for 1 h at RT depending on the source of the primary antibody. Data were obtained using the ChemiDoc XRS+ system (Bio-Rad Laboratories). The band intensities were quantified using Image J. Antibodies used for western blot analysis in this study are as follows: anti-GAPDH (Santa Cruz, sc-365062, 1:3,000), anti-SREBP2 (ImmunoWay, YN0037, 1:1000), anti-SREBP2 (Abcam, ab30682, 1:1000), anti-CYP1A2 (Santa Cruz, sc-53614, 1:1000), goat anti-Rabbit IgG (ZSGB-bio, ZB-2307, 1:5,000), goat anti-Mouse IgG (ZSGB-bio, ZB-2305, 1:500).

### Cell culture

Human primary hepatocytes (Lonza, HUCPI) were cultured as described previously (Xiang et al., 2019). In brief, five chemicals (5C) were added to serum-free medium for long-term hepatocyte culture. The condition contained Williams’ medium E containing B27 (50×, Gibco), Glutamax (Gibco), Pen Strep (Gibco) and five chemicals. Five chemicals: Forskolin (20 μmol/L), SB431542 (10 μmol/L), IWP2 (0.5 μmol/L), DAPT (5 μmol/L), and LDN193189 (0.1 μmol/L). After a period of 5-7 days, the medium was depleted of 5C, followed by a prolonged culture of hepatocytes for 5-8 days, resulting in cellular senescence. No mycoplasma contamination was detected during cell culture.

### Plasmid construction

To generate plasmids encoding SREBP2, cDNA was generated from the pcDNA3.1-2xFLAG-SREBP-2 plasmid (Addgene #26807) by PCR amplification and then cloned into the pLE4 vector (a kind gift from Dr. Tomoaki Hishida) that had been pre-cleaved by *Bam*H1 and *Mlu*I (Deng et al., 2019). The pLE4 vector expressing luciferase (Luc) was used as control. The primer information was listed in Table S11.

### Lentivirus packaging

HEK293T cells were transfected with lentiviral overexpression plasmid and lentiviral packaging vectors psPAX2 (Addgene #12260) and pMD2.G (Addgene #12259). The viral particles were then collected at 48 and 72 h post-transfection, respectively, and concentrated by ultracentrifugation at 19,400×*g* for 2.5 h.

### SA-β-gal staining

For SA-β-gal staining, primary human hepatocytes were fixed in 2% formaldehyde and 0.2% glutaraldehyde for 5 min at RT, washed in PBS, and stained with freshly prepared staining solution until the appropriate time at 37°C (X-gal was purchased from Amresco, all other reagents were from Sigma-Aldrich) (Ren et al., 2019).

### Nucleus isolation and snRNA-seq on the 10× genomics platform

Nucleus isolation was performed using a previously published protocol (Krishnaswami et al., 2016; Ma et al., 2021; Zhang et al., 2021). In short, frozen primate tissue were fully pestled and 1.5 mL lysis buffer were added, which contained 250 mmol/L sucrose, 25 mmol/L KCl, 5 mmol/L MgCl_2_, 10 mmol/L Tris buffer, 1 μmol/L DTT, 1× protease inhibitor, 0.4 U/μL RNaseIn, 0.2 U/μL Superasin, 0.1% Triton X-100, 1 μmol/L propidium iodide (PI), and 10 ng/mL Hoechst 33342 in nuclease-free water. Samples and lysis buffer were gently shaken and mixed, placed on ice for a while, then filtered through a 40 μm cell filter (BD Falcon), centrifuged at 1000 ×*g* for 8 min at 4°C, followed by resuspension in PBS supplemented with 0.3% BSA, 0.4 U/μL RNaseIn and 0.2 U/μL Superasin. Nuclei were labeled using Hoechst 33342 and PI, sorted by FACS (BD Influx), and counted using a dual fluorescence cell counter (Luna-FLTM, Logos Biosystems). The 10× Genomics single-cell 3ʹ system was used for mononuclear capture. Approximately 6,000 nuclei were captured for each sample following the standard 10× capture and library preparation protocol (10× Genomics) and then sequenced in a NovaSeq 6000 sequencing system (Illumina, 20012866).

### Processing of raw data from snRNA-seq and reducing ambient RNA bias

Cell Ranger Single-Cell Software Suite (version 4.0.0) was used for create pre-mRNA reference of Macaca fascicularis 6.0 and calculate gene expression matrices for downstream analyses through the “count” function and the default parameters. CellBender (version 0.2.0) (Fleming et al., 2022) software was used to remove possible background RNA bias with default parameters. 2 samples with too much cell number (expected 10,000 but more than 20,000) and high expression level of background RNA were removed from downstream analyses.

### Filtering of low-quality cells, integration, clustering, and identification of cell types

The R package Seurat (version 4.0.2) (Hao et al., 2021) was used for downstream analyses of filtering low-quality cells, data normalization, dimensionality reduction, clustering, and gene differential expression analysis. Cells with fewer than 200 genes or with mitochondrial gene ratio more than 5% were excluded as low-quality cells. DoubletFinder (version 2.0.3) (McGinnis et al., 2019) software was used for detection and deletion of possible doublets from the technical artifact. After normalization and scaling of the expression matrix for each sample by the function “SCTransfrom”, the “PrepSCTIntegration” and “FindIntegrationAnchors” functions were used to select the features and anchors for downstream integration. All valuable samples were then integrated by function “IntegrateData” according to the features and anchors above-mentioned and scaled by function “ScaleData”. After data integration and scaling, the functions of “RunPCA” and “FindClusters” were used for performing the principle component analysis and clustering. Dimensionality reduction was then performed through the “RunUMAP” function. Cell clustering was performed by “FindNeighbors” and “FindClusters” functions. The function “FindAllMarkers” (avg_log_2_FC ≥ 0.5 and *P*_val_adj ≤ 0.05) was used to calculate marker genes for each cluster. Three clusters in the liver snRNA-seq data, without specific marker genes and have high mitochondrial gene ratios or relatively low gene numbers, were removed due to low quality. By the end of the above steps, 23 clusters of 81,679 nuclei were considered to be of high quality and used for downstream analyses. Nine cell types in liver were identified according to the expression of classical marker genes of each cluster (Table S2). PCA of cell-type-specific markers was analyzed by metascape (Zhou et al., 2019).

### Cell identity score analysis

Gene set represent cell identity of each cell type were calculated by using the function “FindAllMarkers” (avg_log2FC ≥ 0.5 and *P*_val_adj ≤ 0.05) of Seurat to compute top 50 marker genes of each cell type in young group. The function “AddModuleScore” was then used to calculate cell identity score of each cell type in young and aged groups.

### Gene transcriptional noise analysis

To detect the transcriptional fluctuation during aging, the aging-related transcriptional noise in each cell type was analyzed following the previous work (Angelidis et al., 2019). Briefly, cells were down-sampled to equal UMI count size and each cell type were down-sampled to same number of young and aged cells to eliminate the differences of total UMI counts and cell type frequency, respectively. The Euclidean distance between each cell and the corresponding cell type mean within each age group was then calculated and used to measure the transcriptional noise of each cell.

### Aging-related differential expression analysis from snRNA-seq data

Aging-related differentially expressed genes (aging DEGs) between aged and young groups (Aged/Young) were calculated using the Seurat function “FindMarkers” with the Wilcoxon signed-rank test. Genes with | avg_log2FC | ≥ 0.25 and *P*_val_adj ≤ 0.05 were identified as aging DEGs (Table S3).

### Pathway enrichment analysis

Gene Ontology (GO) process and pathway enrichment analysis of aging DEGs were performed by metascape. Kappa-test score (Cohen, 1960) were calculated between each two representative terms selected from the metascape results (*P* value ≤ 0.05) and set as similarity score between terms. Cytoscape (version 3.9.1) was used for visualization of representative GO terms, terms were set as nodes and similarity scores that more than 0.3 were set as edges.

### Gene set score analysis

Gene sets were downloaded from KEGG and GO databases. Gene sets expression scores were calculated using the function “AddModuleScore” of Seurat and visualized through R package ggplot2. Genes in each gene set are listed in Table S9.

### Transcriptional regulatory network analysis

The upstream transcriptional regulatory networks of aging DEGs were analyzed through R packages GENIE3 (version 1.12.0) and RcisTarget (version 1.10.0) of the R package SCENIC (version 1.2.4) (Aibar et al., 2017) workflow with default parameters. Transcription factors (TFs) of hg19 genome was downloaded from RcisTarget database and used as reference. Briefly, TF-genes co-expression networks were firstly constructed through GENIE3 based on the gene expression matrix, where each row represents an aging DEG and each column represents a nucleus, of each cell type, respectively. The enriched transcription factor-binding motifs and their target genes (regulons) were then inferred through RcisTarget. The transcriptional regulatory module network was visualized through Cytoscape (version 3.9.1).

### Cell–cell communication analysis

CellPhoneDB software (version 1.1.0) (Efremova et al., 2020) was used to infer the intercellular communication network from the snRNA-seq data. Only receptors and ligands expressed in more than 10% of cells of a certain cell type from the young or aged groups were retained in further analysis. The average expression of each ligand-receptor pair was calculated between each pair of cell types, and only those with *P* values ≤ 0.05 were considered as the available prediction of the cell–cell communications (Table S4).

### Aging sensibility analysis

The prioritization of the most responsive cell types during monkey liver aging was calculated using the function “calculate_auc” of R package Augur (version 1.0.3) by inputting the seurat object with “cell type” and “age group” labels.

### Bulk RNA-seq data processing

Fastp (version 0.23.2) software was used for quality control, adapter trimming, quality filtering of raw bulk RNA-seq reads. HISAT2 (version 2.0.4) (Kim et al., 2015) was then used for mapping the trimmed reads to the Macaca fascicularis 6.0 genome. The generated sam files were then converted to bam files through SAMtools (version 1.6). The read count of each gene was calculated through the featureCounts (version 2.0.3) software. R package DESeq2 (version 1.2.4) (Love et al., 2014) was used to identify DEGs between aged and young samples (Aged/Young) with the cutoff values of adjusted *P* value ≤ 0.05 and |Log_2_FC | ≥ 0.5.

### Statistical analysis

The statistical analysis was performed and analyzed by two-tailed Student’s *t*-test in Graphpad Prism 8.0 software. Data are presented as the mean ± SEM. Differences were considered significant when *P* < 0.05. *P < 0.05, **P < 0.01, ***P < 0.001.

## Supplementary Material

pwad039_suppl_Supplementary_Materials

pwad039_suppl_Supplementary_Tables

## Data Availability

All data associated with this study are present in the paper or the Supplementary Materials. The original sequencing data in this study have been deposited in the Genome Sequence Archive in the National Genomics Data Center, Beijing Institute of Genomics (China National Center for Bioinformation) of the Chinese Academy of Sciences, with accession number CRA010436.

## Abbreviations

BC: B cells
BMI: body mass index
Cho: cholangiocytes
CYPs: cytochrome P450 proteins
DAMP: damage-associated molecular patterns
DEGs: differentially expressed genes
EC: endothelial cells
ECM: extracellular matrix
FACS: fluorescence-activated cell sorting
GO: gene ontology
HE: hematoxylin–eosin
Hep: hepatocytes
HSCs: hepatic stellate cells
IL: interleukin
Kup: Kupffer cells
LDs: lipid drops
Log2FC: Log2 fold change
LSECs: liver sinusoidal endothelial cells
MPO: myeloperoxidase
Myof: myofibroblasts
NAD: nicotinamide adenine dinucleotide
NHPs: non-human primates
NPCs: non-parenchyma cells
PBS: phosphate buffer solution
PCA: principal component analysis
PFA: paraformaldehyde
PI: propidium iodide
RT: room temperature
RT-qPCR: real-time quantitative reverse transcription polymerase chain reaction
SA-β-gal: senescence-associated β-galactosidase
SBB: Sudan black B
SCENIC: single-cell regulatory network inference and clustering
scRNA-seq: single-cell RNA sequencing
SMC: smooth muscle cells
snRNA-seq: single-nucleus RNA sequencing
SREBPs: sterol regulatory element-binding proteins
TC: T cells
TFs: transcription factors
TGF-β: transforming growth factor-β
t-SNE: t-distributed stochastic neighbor embedding
TUNEL: TdT (terminal deoxynucleotidyl transferase)-mediated dUTP nick-end labeling
UMAP: uniform manifold approximation and projection
UMI: unique molecular identifier.

## Code availability

No code or model was generated in this study.
