# Supplementary material for: A single-nucleus transcriptomic atlas of primate liver aging uncovers the pro-senescence role of SREBP2 in hepatocytes
Source: Protein Cell. 2023 Jun 28;15(2):98–120. doi: 10.1093/procel/pwad039 (PMC10833472; doi:10.1093/procel/pwad039)
Supplement: pwad039_suppl_Supplementary_Materials [file pwad039_suppl_supplementary_materials.pdf]

Supplemental Materials

Supplemental Figure Legends

Figure S1. The phenotypic characterizations and snRNA-seq data quality control in cynomolgus monkey livers.

(A) Information on the monkeys used in this study.

(B) BMI index of young and aged monkey livers. Data are quantified as fold changes and shown as means ± SEM. Young, n = 8; aged, n = 8. ns, no significance.

(C) The ratio of liver weight to whole body weight in young and aged monkey livers. Data are quantified as fold changes and shown as means ± SEM. Young, n = 8; aged, n = 8. ns, no significance.

(D) Scatter plot showing the PCA distribution of bulk RNA-seq expression matrix among samples isolated from 16 monkeys.

(E) Heatmap showing the Euclidean distance of bulk RNA-seq expression matrix among samples isolated from 16 monkeys.

(F) Scatter plot showing the gene counts, UMI counts and proportion of mitochondrial genes of each nucleus. The color key from dark to light indicates less to more percentage of mitochondrial genes. Low quality nuclei are colored by black.

(G) Boxplots showing the number of UMI (left) and gene (right) detected in each sample.

(H) UMAP plots showing the distribution of nuclei from each liver sample.

(I) UMAP plots showing the distribution of cluster for nuclei from all the liver samples.

(J) Violin plots showing the cell identity score of each cell type from young and aged liver samples.

(K) Box plots showing cell-cycle proportion of hepatocytes in young and aged liver samples.

Figure S2. snRNA-seq analysis for changes in cell-type-specific gene expression and cell-cell interactions.

(A) Heatmap showing the representative GO terms and pathways of upregulated (left) and downregulated (right) aging-related DEGs across cell types.

(B) Circos plots visualizing the young group-specific (top) and aged group-specific (bottom) cell-cell interactions across cell types. Each curve that connects a ligand and a receptor

represents an interaction.

(C) Bar plots showing the representative GO terms and pathways of young group-specific (left) and aged group-specific (right) cell-cell interaction pairs.

Figure S3. Database collaborative analysis and transcriptional characterization of hepatocyte subtypes.

(A) Dot plot showing the representative GO terms and pathways of genes in figure 4G.

(B) Heatmap (left) showing the overlapped DEGs between snRNA-seq and bulk RNA-seq, and heatmap (right) showing the corresponding functional annotations.

(C) Ring heatmap showing the overlapped DEGs between snRNA-seq and bulk RNA-seq with the circles representing the DEGs of bulk RNA-seq. The color key of heatmap from blue to red indicates the Log_2FC of DEGs in snRNA-seq from low to high. The size of circles shows the $|Log_2FC|$ of DEGs in bulk RNA-seq. The circle colors of red and blue represent upregulated and downregulated genes in bulk RNA-seq respectively.

(D) Dot plot showing the expression level of representative marker genes across hepatocyte subtypes. The color key from gray to red presents gene expression level from low to high. The size of dots indicates the percentage of cells with gene expression greater than 0.

(E) Arc plot showing prioritization of hepatocyte subtypes during aging.

(F) Violin plot showing the zonation-specific metabolic characteristics of each hepatocyte subtype. The blue, orange and black colors of *P* values represent compared groups of young samples to young samples, aged samples to aged samples, aged samples to young samples. The dotted lines of each pathway are drawn with the lowest mean of the gene sets score across 3 hepatocyte subtypes in young and aged groups.

(G) Network showing the representative GO terms and pathways of upregulated *SREBP2* target genes in hepatocytes from young and aged monkey livers.

(H) RT-qPCR analysis for mRNA levels of genes related to cholesterol biosynthesis in human primary hepatocytes transduced with lentiviruses expressing SREBP2 or Luc. Data are quantified as fold changes and shown as means ± SEM, $n = 3$ biological replicates. $^*P < 0.05$, $^{**}P < 0.01$.

Supplementary Table Legends

Table S1. Differentially expressed genes (DEGs) of bulk RNA-seq between aged and young livers.

Table S2. Marker genes of each cell type in cynomolgus monkey livers.

Table S3. DEGs of each cell type between aged and young livers.

Table S4. Young and aged group-specific cell-cell interactions across cell types in cynomolgus monkey livers.

Table S5. List of genes annotated in liver disease-related gene sets.

Table S6. List of genes annotated in Aging Atlas database.

Table S7. List of proteins annotated in aging-associated serum proteins.

Table S8. Marker genes of each hepatocyte subtype.

Table S9. List of genes annotated in hepatic functional gene sets.

Table S10. Aging DEGs of hepatocyte subtypes in cynomolgus monkey livers.

Table S11. Primers used in this study.

Figure S1

A

Gender	Sample number	Age (Year)	Sample number	Age (Year)	Gender	Sample number	Age (Year)	Sample number	Age (Year)
Male	YM1	5	OM1	18	Female	YF1	5	OF1	18
Male	YM2	5	OM2	19	Female	YF2	5	OF2	19
Male	YM3	6	OM3	20	Female	YF3	5	OF3	19
Male	YM4	6	OM4	21	Female	YF4	4	OF4	20

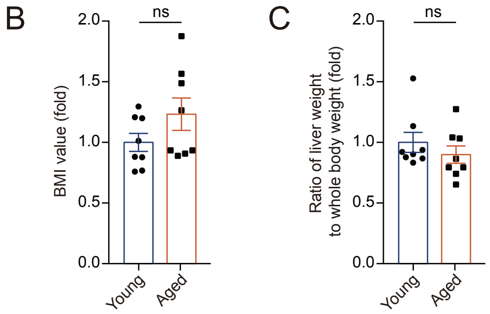

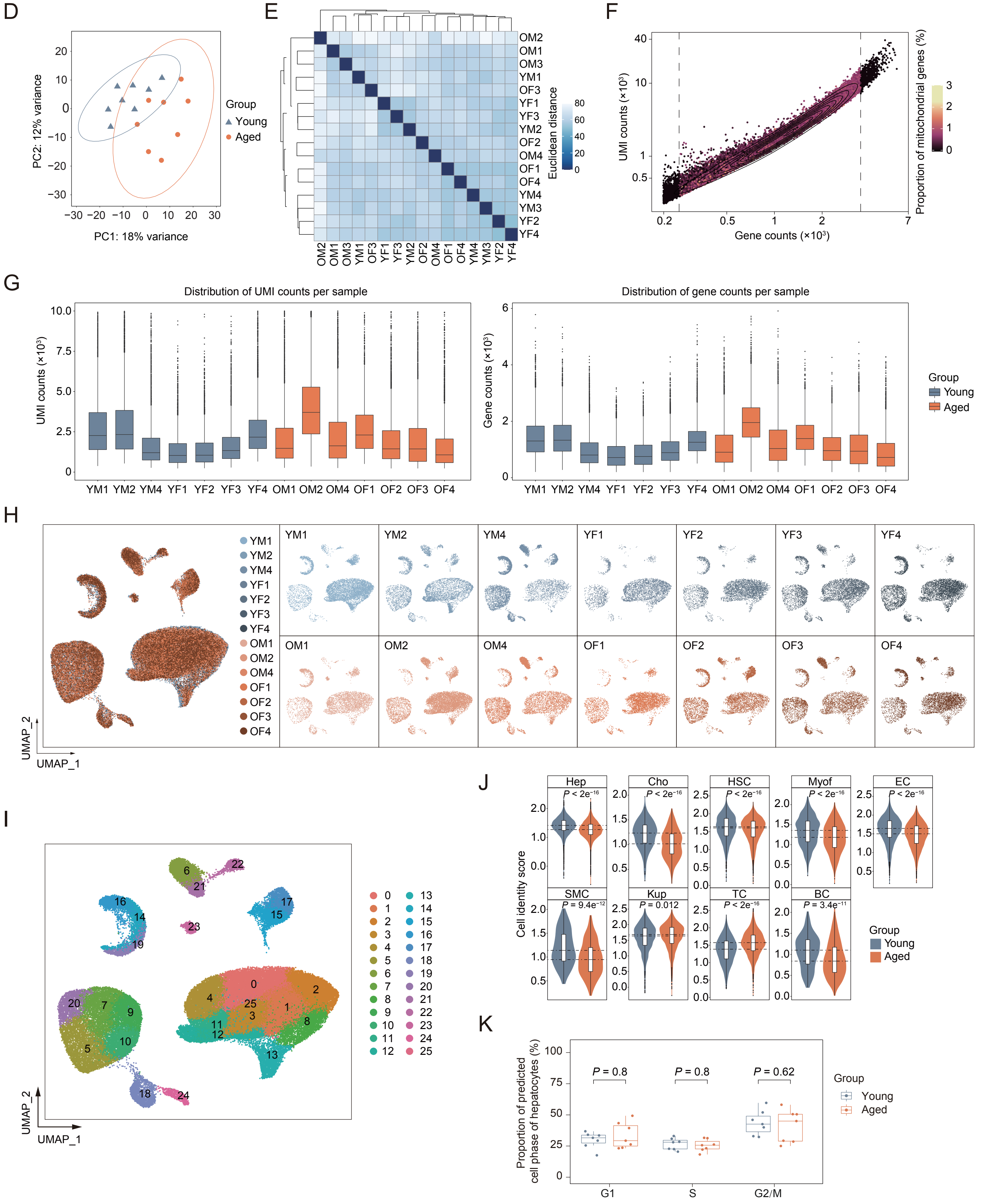

Figure S2

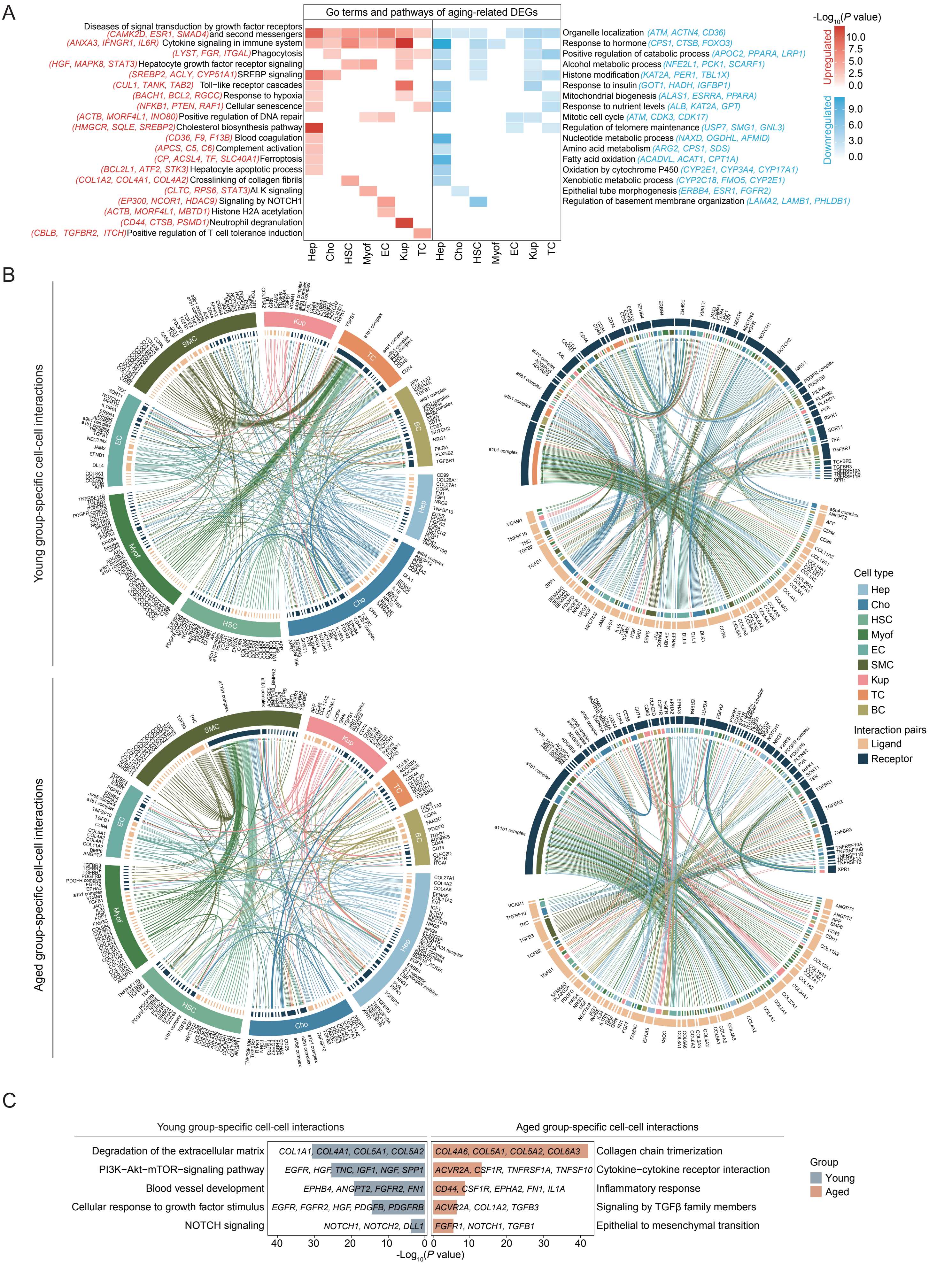
A
Go terms and pathways of aging-related DEGs
Diseases of signal transduction by growth factor receptors (CAMK2D, ESR1, SMAD4) and second messengers
(ANXA3, IFNGR1, IL6R) Cytokine signaling in immune system
(LYST, FGR, ITGAL) Phagocytosis
(HGF, MAPK8, STAT3) Hepatocyte growth factor receptor signaling
(SREBP2, ACLY, CYP51A1) SREBP signaling
(CUL1, TANK, TAB2) Toll−like receptor cascades
(BACH1, BCL2, RGCC) Response to hypoxia
(NFKB1, PTEN, RAF1) Cellular senescence
(ACTB, MORF4L1, INO80) Positive regulation of DNA repair
(HMGCR, SQLE, SREBP2) Cholesterol biosynthesis pathway
(CD36, F9, F13B) Blood coagulation
(APCS, C5, C6) Complement activation
(CP, ACSL4, TF, SLC40A1) Ferroptosis
(BCL2L1, ATF2, STK3) Hepatocyte apoptotic process
(COL1A2, COL4A1, COL4A2) Crosslinking of collagen fibrils
(CLTC, RPS6, STAT3) ALK signaling
(EP300, NCOR1, HDAC9) Signaling by NOTCH1
(ACTB, MORF4L1, MBTD1) Histone H2A acetylation
(CD44, CTSB, PSMD1) Neutrophil degranulation
(CBLB, TGFBR2, ITCH) Positive regulation of T cell tolerance induction
Organelle localization (ATM, ACTN4, CD36)
Response to hormone (CPS1, CTSB, FOXO3)
Positive regulation of catabolic process (APOC2, PPARA, LRP1)
Alcohol metabolic process (NFE2L1, PCK1, SCARF1)
Histone modification (KAT2A, PER1, TBL1X)
Response to insulin (GOT1, HADH, IGFBP1)
Mitochondrial biogenesis (ALAS1, ESRRA, PPARA)
Response to nutrient levels (ALB, KAT2A, GPT)
Mitotic cell cycle (ATM, CDK3, CDK17)
Regulation of telomere maintenance (USP7, SMG1, GNL3)
Nucleotide metabolic process (NAXD, OGDHL, AFMID)
Amino acid metabolism (ARG2, CPS1, SDS)
Fatty acid oxidation (ACADVL, ACAT1, CPT1A)
Oxidation by cytochrome P450 (CYP2E1, CYP3A4, CYP17A1)
Xenobiotic metabolic process (CYP2C18, FMO5, CYP2E1)
Epithelial tube morphogenesis (ERBB4, ESR1, FGFR2)
Regulation of basement membrane organization (LAMA2, LAMB1, PHLDB1)
Hep Cho HSC Myof EC Kup TC Hep Cho HSC Myof EC Kup TC
-Log10(P value)
Upregulated 10.0 7.5 5.0 2.5 0.0
Downregulated 9.0 6.0 3.0 0.0
B
Young group-specific cell-cell interactions
Aged group-specific cell-cell interactions
Cell type
Hep
Cho
HSC
Myof
EC
SMC
Kup
TC
BC
Interaction pairs
Ligand
Receptor
C
Young group-specific cell-cell interactions
Aged group-specific cell-cell interactions
Degradation of the extracellular matrix — COL1A1, COL4A1, COL5A1, COL5A2
PI3K−Akt−mTOR−signaling pathway — EGFR, HGF, TNC, IGF1, NGF, SPP1
Blood vessel development — EPHB4, ANGPT2, FGFR2, FN1
Cellular response to growth factor stimulus — EGFR, FGFR2, HGF, PDGFB, PDGFRB
NOTCH signaling — NOTCH1, NOTCH2, DLL1
COL4A6, COL5A1, COL5A2, COL6A3 — Collagen chain trimerization
ACVR2A, CSF1R, TNFRSF1A, TNFSF10 — Cytokine−cytokine receptor interaction
CD44, CSF1R, EPHA2, FN1, IL1A — Inflammatory response
ACVR2A, COL1A2, TGFB3 — Signaling by TGFβ family members
FGFR1, NOTCH1, TGFB1 — Epithelial to mesenchymal transition
40 30 20 10 0 0 10 20 30 40
-Log10(P value)
Group
Young
Aged

Figure S3

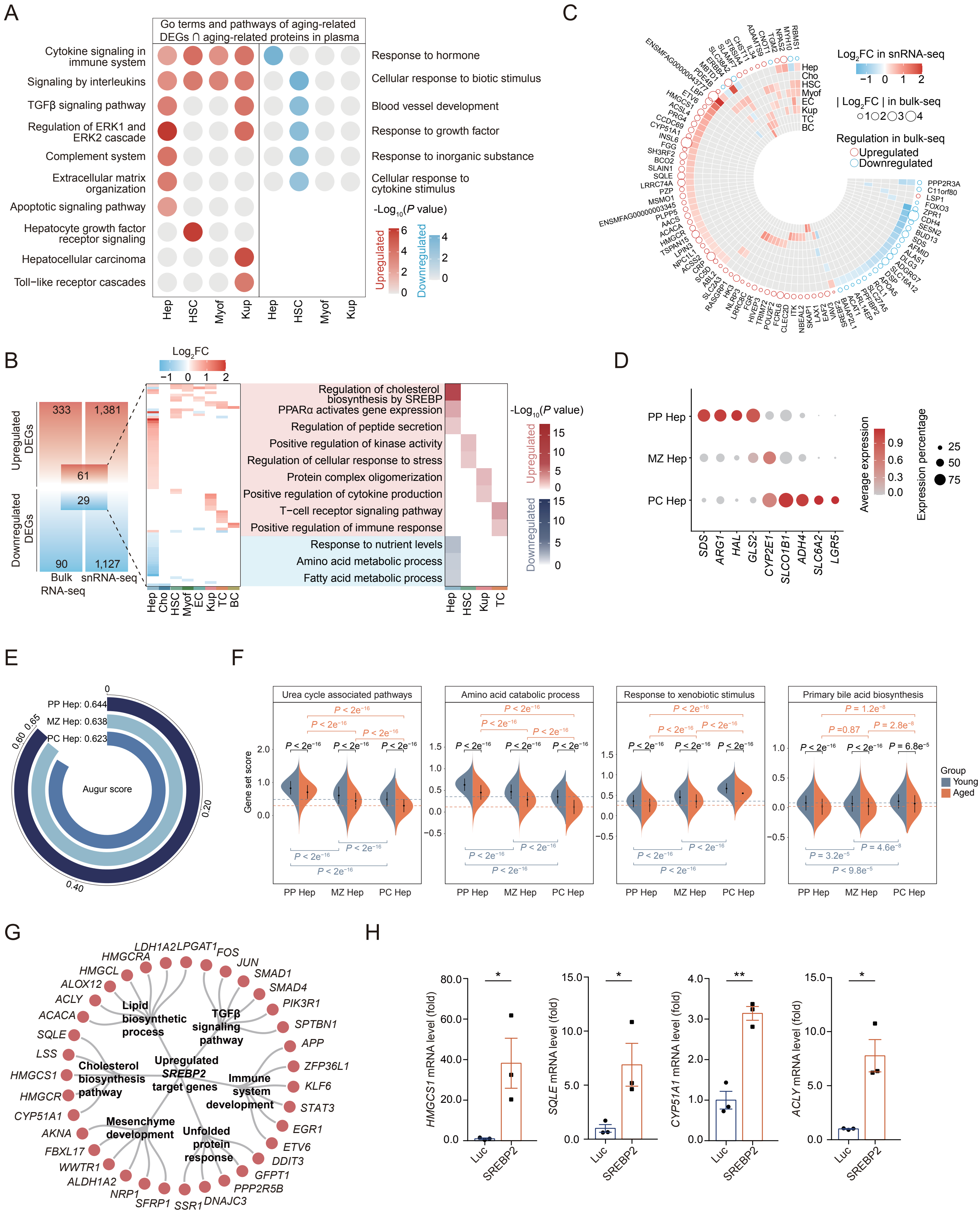
A
Go terms and pathways of aging-related DEGs ∩ aging-related proteins in plasma
Cytokine signaling in immune system
Signaling by interleukins
TGFβ signaling pathway
Regulation of ERK1 and ERK2 cascade
Complement system
Extracellular matrix organization
Apoptotic signaling pathway
Hepatocyte growth factor receptor signaling
Hepatocellular carcinoma
Toll−like receptor cascades
Response to hormone
Cellular response to biotic stimulus
Blood vessel development
Response to growth factor
Response to inorganic substance
Cellular response to cytokine stimulus
-Log10(P value)
Upregulated
Downregulated
Hep HSC Myof Kup Hep HSC Myof Kup
B
Log2FC
Upregulated DEGs
Downregulated DEGs
333 1,381 61 29 90 1,127
Bulk RNA-seq snRNA-seq
Regulation of cholesterol biosynthesis by SREBP
PPARα activates gene expression
Regulation of peptide secretion
Positive regulation of kinase activity
Regulation of cellular response to stress
Protein complex oligomerization
Positive regulation of cytokine production
T−cell receptor signaling pathway
Positive regulation of immune response
Response to nutrient levels
Amino acid metabolic process
Fatty acid metabolic process
-Log10(P value)
Hep Cho HSC Myof EC Kup TC BC
Hep HSC Kup TC
C
Log2FC in snRNA-seq
| Log2FC | in bulk-seq
Regulation in bulk-seq
Upregulated
Downregulated
Hep Cho HSC Myof EC Kup TC BC
D
PP Hep
MZ Hep
PC Hep
SDS ARG1 HAL GLS2 CYP2E1 SLCO1B1 ADH4 SLC6A2 LGR5
Average expression
Expression percentage
E
PP Hep: 0.644
MZ Hep: 0.638
PC Hep: 0.623
Augur score
F
Urea cycle associated pathways
Amino acid catabolic process
Response to xenobiotic stimulus
Primary bile acid biosynthesis
Gene set score
Group
Young
Aged
PP Hep MZ Hep PC Hep
G
Upregulated SREBP2 target genes
Lipid biosynthetic process
TGFβ signaling pathway
Cholesterol biosynthesis pathway
Immune system development
Mesenchyme development
Unfolded protein response
H
HMGCS1 mRNA level (fold)
SQLE mRNA level (fold)
CYP51A1 mRNA level (fold)
ACLY mRNA level (fold)
Luc SREBP2
